# An elm EST database for identifying leaf beetle egg-induced defense genes

**DOI:** 10.1186/1471-2164-13-242

**Published:** 2012-06-15

**Authors:** Kerstin Büchel, Eric McDowell, Will Nelson, Anne Descour, Jonathan Gershenzon, Monika Hilker, Carol Soderlund, David R Gang, Trevor Fenning, Torsten Meiners

**Affiliations:** 1 Freie Universität Berlin, Applied Zoology / Animal Ecology, Berlin, Germany; 2 Max Planck Institute for Chemical Ecology, Dept. of Biochemistry, Jena, Germany; 3 Washington State University, Institute of Biological Chemistry, Pullman, WA, USA; 4 University of Arizona, BIO5 Institute and Plant Sciences, Tucson, AZ, USA; 5 Forest Research, Northern Research Station, Midlothian, Scotland, UK

## Abstract

**Background:**

Plants can defend themselves against herbivorous insects prior to the onset of larval feeding by responding to the eggs laid on their leaves. In the European field elm (*Ulmus minor)*, egg laying by the elm leaf beetle ( *Xanthogaleruca luteola*) activates the emission of volatiles that attract specialised egg parasitoids, which in turn kill the eggs. Little is known about the transcriptional changes that insect eggs trigger in plants and how such indirect defense mechanisms are orchestrated in the context of other biological processes.

**Results:**

Here we present the first large scale study of egg-induced changes in the transcriptional profile of a tree. Five cDNA libraries were generated from leaves of (i) untreated control elms, and elms treated with (ii) egg laying and feeding by elm leaf beetles, (iii) feeding, (iv) artificial transfer of egg clutches, and (v) methyl jasmonate. A total of 361,196 ESTs expressed sequence tags (ESTs) were identified which clustered into 52,823 unique transcripts (Unitrans) and were stored in a database with a public web interface. Among the analyzed Unitrans, 73% could be annotated by homology to known genes in the UniProt (Plant) database, particularly to those from *Vitis*, *Ricinus*, *Populus* and *Arabidopsis*. Comparative *in silico* analysis among the different treatments revealed differences in Gene Ontology term abundances. Defense- and stress-related gene transcripts were present in high abundance in leaves after herbivore egg laying, but transcripts involved in photosynthesis showed decreased abundance. Many pathogen-related genes and genes involved in phytohormone signaling were expressed, indicative of jasmonic acid biosynthesis and activation of jasmonic acid responsive genes. Cross-comparisons between different libraries based on expression profiles allowed the identification of genes with a potential relevance in egg-induced defenses, as well as other biological processes, including signal transduction, transport and primary metabolism.

**Conclusion:**

Here we present a dataset for a large-scale study of the mechanisms of plant defense against insect eggs in a co-evolved, natural ecological plant–insect system. The EST database analysis provided here is a first step in elucidating the transcriptional responses of elm to elm leaf beetle infestation, and adds further to our knowledge on insect egg-induced transcriptomic changes in plants. The sequences identified in our comparative analysis give many hints about novel defense mechanisms directed towards eggs.

## Background

Trees grow under a multitude of abiotic and biotic stresses. Although the suite of genes in trees is similar to that in herbaceous and crop plants, the ecological survival strategies of trees and especially the regulation mechanisms of their secondary metabolic processes are likely to differ from those of herbaceous plants, because of the different life times and size of these types of plants [1-4]. The advent of high-throughput sequencing technologies enables a broad snapshot of the molecular-genetic processes in plant, and have already been used to reveal the large scale transcriptional alterations that occur in plant–insect interactions [5,6]. However, most of the current knowledge about plant defense mechanisms against herbivorous insects has been obtained from studies with herbaceous annuals or short-lived perennials, with few studies of the modulation of complex tree defensive responses.

From an ecological and evolutionary research perspective, the optimal tree species for studying defense mechanisms would be one that has been unaffected by breeding for agriculture and forestry, and that is attacked by a highly specialized pest organism. Such conditions can be found for the field elm (*Ulmus minor)* and its closely co-evolved herbivore, the elm leaf beetle ( *Xanthogaleruca luteola*) [7,8].

Plants have developed various mechanisms to defend themselves against herbivorous insects [9,10]. In addition to nonspecific, constitutively expressed physical and chemical barriers (e.g. trichomes, thick cell walls, adverse secondary metabolites), plants employ specific induced defenses in response to insect feeding or even egg laying [11,12].

In contrast to feeding, insect egg laying causes minimal damage to plants, dependent on the egg laying behavior of herbivorous insects, which can be quite distinct in different species [13,14]. Direct defenses against insect eggs have been reported for crop and herbaceous species including the production of ovicidal substances [15], growth of neoplasms [16], development of necrotic zones [17,18]. Indirect defense against insect egg laying includes induced changes of plant volatile emissions or modifications of the plant surface chemistry attracting or arresting egg parasitoids, which in turn kill the eggs of the herbivores [19,20].

The first study demonstrating indirect defense against insect eggs was a study of the field elm, where eggs of the elm leaf beetle induced volatiles which attract the egg parasitoid *Oomyzus gallerucae,* a tiny eulophid wasp specialized on elm leaf beetle eggs [21]. Elm leaf beetles often feed and lay eggs on the same plant and are known to remove the leaf epidermis prior to egg laying by scratching the leaf surface with their mouthparts. Experimental simulation of this egg laying sequence by transferring eggs or oviduct secretion on scratched elm leaves or treatment with jasmonic acid (JA) or methyl jasmonate (MeJA) also elicited indirect defense responses in field elms ([8,21], Meiners T. unpublished data). A recent study further showed that terpenoids present in the odor of egg-induced elm leaves are relevant for attraction of the egg parasitoids [22]. Induction of attractive plant volatiles by insect egg laying has been shown in one other tree species and two herbaceous crops [8,23-25].

The natural range of the European field elm *Ulmus minor* (Ulmaceae) extends predominantly within Southern Europe. However, through cultivation it occurs throughout the temperate world. Elms are greatly valued for their timber qualities and prior to the Dutch elm disease outbreaks, elms were also frequently planted within urban areas because of their environmental tolerance [26,27]. Many insects including moths, gall mites, and beetles feed on field elms. The elm leaf beetle *X. luteola* can defoliate entire trees and is recognized as a major urban and forest pest in the USA and Australia [28,29]. The recently published EST sequences for *U. americana* is to our knowledge, the only other gene expression study of any *Ulmus* species, where 535 ESTs (grouped into 314 unique transcripts) were identified after trees (hard calli) were exposed to the fungal pathogen *Ophiostoma novo-ulmi*, which is the causative agent of Dutch elm disease [30].

Knowledge on how plants are able to respond at the molecular level towards egg laying is scarce. Specific transcriptional changes of a wide range of genes involved in several metabolic processes have been shown in Brussels sprouts (*Brassica oleracea* var. *gemmifera*) and *Arabidopsis thaliana* in response to *Pieris brassicae* egg laying [31,32]. The formation of neoplasms on pea pods after egg laying by bruchid beetles is associated with the upregulation of genes *inter alia* encoding enzymes involved in the octadecanoid pathway [33]. Scots pine (*Pinus sylvestris*) responds to eggs laid by the pine sawfly by enhancing the transcription of sesquiterpene synthase genes [34].

Inducible defenses might start with the perception of insect attack by the plants. Compounds released onto the leaves by the female insect with her eggs (e.g. oviduct secretion or accessory glandular secretion attaching the eggs to leaf tissue) or substances released into plant wounds during feeding (saliva- or regurgitate-derived compounds) most likely convey the information indicating an “insect attack”, and so trigger a cascade of plant reactions, followed by downstream signaling pathways that mediate specific gene expression leading to the biosynthesis of metabolites which are responsible for the direct and indirect defenses [11,35].

It has been suggested that plants orchestrate their defense reactions against different insect herbivores by a cross-talk between phytohormone pathways, with the octadecanoid signal-transduction pathway playing a key role in this process [36-38]. However, although jasmonic acid (JA) is known to induce indirect defenses in plants *via* the production of volatiles that attract egg parasitoids, the headspace profiles of egg-induced plants and JA-treated ones differ from each other indicating that other plant hormones are also involved in the orchestration of defenses that signal the presence of eggs to egg parasitoids [39,40].

Herbivore eggs have been shown to induce changes in the plant’s primary and secondary metabolism and can cause dramatic changes in the plant’s transcriptome [31,32]. To date, however, only two studies of Scot pine and Brussels sprouts have addressed the role of egg-induced transcriptional changes in indirect defenses [32,34,41].

We have shown previously that elms can produce a distinct eco-physiological response to the egg laying activities of elm leaf beetle even in the absence of herbivory [8]. The elegant subtlety of these responses and the co-evolved species specificity predestinate this natural ecological *U. minor* - *X. luteola - O. gallerucae* system for studying egg-induced transcriptional changes in plants. Here we present the first time a large-scale study of insect egg-induced defense in a natural ecological plant–insect-system.

For identification of egg-induced genes in the field elm, five cDNA libraries were constructed from young elm trees of a single clone. Leaves were harvested after different time periods and different treatments with feeding and/or egg laying by the elm leaf beetle, artificial transfer of egg clutches (to distinguish between egg laying and feeding effects), and spraying with MeJA. A total of 361,196 expressed sequence tags (ESTs) were pyro-sequenced and assembled into unique transcripts (Unitrans). Here we report the comparative analysis of 21,490 Unitrans (each represented by at least two ESTs) in order to detect differences in functionally annotated gene transcript abundances. This EST collection represents the first large genomic resource for the European field elm, and the database is now available with a public web interface (http://www.agcol.arizona.edu/pave/elm), where it is possible to query the different elm libraries based on ESTs, Unitrans, UniProt IDs / descriptions, Protein Families (Pfam), Enzyme Commission numbers (EC) and Gene Ontology terms (GO).

## Results

### Sequencing of elm after treatment with leaf beetles

Non-normalized total RNA was isolated from leaves of clonal *U. minor* plants that had been exposed to one of five separate treatments: untreated intact elm leaves (C = control), leaves with egg laying and feeding by the elm leaf beetle, *Xanthogaleruca luteola* (EF), leaves with feeding alone by adult *X. luteola* (F), scratched leaves (removal of leaf epidermis to mimic natural egg laying) with manually transferred egg clutches to the scratched site (E); and leaves sprayed with methyl jasmonate (MeJA). Random cDNAs were synthesized from each of these mRNA samples and 454 pyrosequenced. An additional three samples, consisting of mixtures of cDNA libraries, were also sequenced to increase sequence coverage for detected genes (Table 1). After pre-processing, clustering and assembling, we obtained 21,490 Unitrans (unique transcripts) represented by at least two ESTs plus 31,333 Unitrans (singletons) represented by one EST to give a total of 52,823 Unitrans. The elm sequencing libraries obtained from the single treatments contained between 811 Unitrans (≥ 2 EST) (E) and 2,272 Unitrans (≥2 EST) (MeJA), with ~20% singletons per library, while for the mixed libraries between, 12,402 Unitrans (≥2 EST) (E) and 15,083 Unitrans (≥2 EST) (EF + F) were obtained with ~40% singletons per library. As is typical for singletons derived from 454 sequencing, many appeared to represent real gene transcripts, whereas the origin of others is questionable and may well be artifacts. For further analysis Unitrans whose sequence quality was sufficient (plant UniProt annotated with E-value ≤1e-20 threshold) were used. A total of 60% of the Unitrans were between 200–400 nt in length and 71% consist of 2–5 ESTs (see Additional files 1 and 2). Most Unitrans (≥2 EST) showed an open reading frame size in the range of 51-100 (singletons 1-50) (Additional file 3). Thus, although this is the first large-scale sequencing project for this genus, it is almost certainly not a complete representation of all genes expressed in these tissues.

**Table 1:** Sequencing output of elm libraries

**cDNA Libraries**^**a**^	**ESTs**	**Unitrans**^**b**^ ≥ 2 ESTs	**Library specific Unitrans**^**c**^	**Singletons (%)**^**d**^
Untreated control (C)	2132	836	31	174 (17)
Egg & feeding (EF)	1921	826	50	211 (20)
Feeding (F)	4725	1453	65	326 (18)
Methyl jasmonate (MeJA)	7080	2272	153	679 (23)
Transferred eggs (E)	2133	811	40	188 (19)
Mix^e ^ EF + F	169672	15083	2844	11560 (43)
C + MeJA + E	71239	9141	860	8043 (47)
C + EF + F + MeJA + E	98210	12402	2249	9755 (44)
Tag unidentifiable	4084	597	200	397 (40)
**Total**	**361196**	**21490**	**–**	**31333 (59)**

^**a**^ Libraries of differently treated elms: C = Control (untreated *Ulmus minor* leaves); EF = Egg laying & feeding (=leaves with elm leaf beetle eggs and feeding damage by female beetles [natural situation]; F = Feeding (=leaves with feeding damage by male beetles); Methyl jasmonate (= leaves treated with 50 μmol MeJA), E = Transferred eggs (= leaves that had been scratched and had eggs artificially placed on them ); ^**b**^ Number of Unitrans (unique transcripts) ≥2 ESTs that contain at least one EST of one of the libraries; ^**c**^ Number of Unitrans ≥2 ESTs that contain ESTs of only the particular library; ^**d**^ Percent singletons in relation to all unique transcripts (Unitrans ≥2 ESTs + singletons); ^**e**^ New sequencing runs of combined libraries.

### Functional annotation of sequenced transcripts

**Figure 1:**
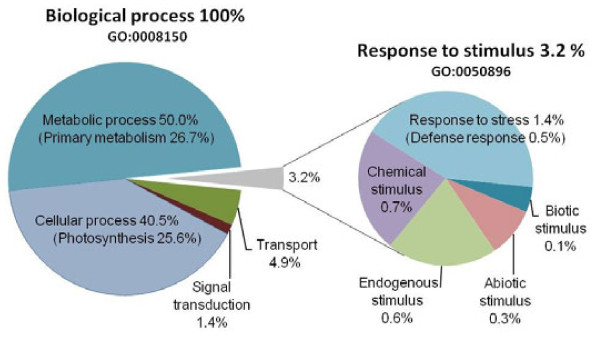
**Functional distribution of all annotated Unitrans (>2 ESTs) based on their predicted Gene Ontology (GO) term.** All GOs shown had at least 200 ESTs and an E value threshold of ≤1e-40 after annotation by the UniProt database. The left chart represents the five main groups of biological processes (GO first level), the right chart represents subcategories for “Response to stimulus” (GO second level), and “Response to stress” (in brackets, GO third level).

Among the total number of Unitrans ≥2 ESTs (21,490), 8,780 (41%) were annotated using BLASTx against the plant taxonomic database of the UniProt protein function and sequence database platform with an E-value threshold of ≤1e-20. Not surprisingly, the most abundant gene products with known function in the elm leaf EST database included genes involved in photosynthesis (Additional file 4). The top four plant genera to which 73% of the Unitrans were annotated using the Plant UniProt database included *Vitis, Ricinus, Populus* and *Arabidopsis* (Additional file 5). The resulting annotated Unitrans were grouped into nine different functional categories based on their Gene Ontology term (GO term, Figure 1). Most Unitrans belonged to the categories “cellular process or metabolic process” (90.5%), whereas 0.5% fell into the category “defense response”.

### Changes in transcript abundances among treatments

The sequencing was performed with the aim of detecting leaf beetle egg-induced defense genes and associated regulatory elements, based on the assumption that changes in abundances of mRNA species are reflected by differences in the number of ESTs that encode particular genes. It is possible for abundances of a given transcript to be falsely low in a sequenced library due to poor quality sequence, insufficient sequence depth, misassembled Unitrans or misidentification of the best organism match for a Unitrans due to sequencing/assembly errors. Hence the R statistic was applied to the elm database and used as an initial statistical screening tool [42]. The library counts were displayed as parts per 10,000 (pptt) or parts per 1,000 (ppt), which normalizes transcript abundances based on their library size. This prevents over-evaluation of high transcript numbers in a large library relative to low numbers of transcript in a smaller library.

**Figure 2:**
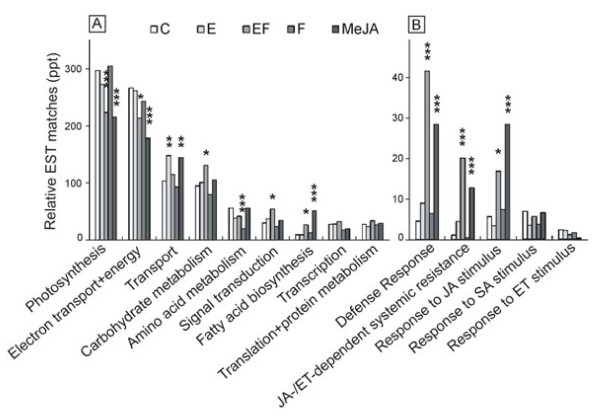
**Functional distribution of elm leaf EST matches from the five different single libraries based on their predicted Gene Ontology (GO) term.** All GOs shown had at least 200 ESTs and an E value threshold of ≤1e-40 after annotation by the UniProt database. The GO terms from each library are divided into two groups: (A) important plant physiological processes and (B) defense-related processes including response to jasmonic acid (JA), ethylene (ET) and salicylate (SA). The libraries are: EF (egg laying & feeding), F (feeding), MeJA (methyl jasmonate), E (artificial scratching & egg transfer) and C (untreated control). The y-axis indicates relative EST matches by parts per thousand (ppt) relative to the library size. For statistical analysis EST abundances by library were compared pairwise by GO category. Asterisks denote treatments in which ESTs were differentially expressed relative to the control treatment; *P ≤ 0.05, **P ≤ 0.01, ***P ≤ 0.001; Fisher`s exact test.

The five treatments were compared using relative EST abundance per annotated GO functional category (i.e., summed across all Unitrans annotated to that category). To obtain a broad overview of the transcriptomic responses in major plant physiological processes, nine GO categories were selected and four of them were considered as significantly differentially expressed in the respective treatment compared to untreated elms (C) (Figure 2a). For the GO term categories “photosynthesis” and “electron transport + energy”, the comparison indicated a decrease in transcript abundances for egg-induced (EF) as well as MeJA treated plants. Chlorophyll a-b binding proteins (Unitrans: elm_00108, data not shown) were mostly responsible for the differential transcript abundances between treatments. For almost all categories, MeJA treated plants showed transcript abundance patterns similar to EF treated plants, suggesting that MeJA does indeed play a significant role in the plant’s response to egg laying. Likewise, similar patterns of transcript abundances were observed between untreated plants (C), feeding-induced plants (F), and plants with the experimental imitation of the egg laying event by transfer of egg clutches (E). For the category “transport” E and MeJA treated plants showed increased transcript levels in comparison to the other treatments. Feeding-induced plants showed decreased transcript levels in comparison to the other treatments only for the category “amino acid metabolism”. In “carbohydrate metabolism” and “signal transduction” a significant increase in transcriptional changes was determined only for egg-induced plants. For these categories no single Unitrans is responsible for the changed transcript pattern. For the category “fatty acid biosynthesis”, the largest group of ESTs responsible for differences between treatments matched a lipoxygenase (Unitrans: elm_00084, data not shown), which is a key enzyme in JA biosynthesis. The strongest increase of lipoxygenase-related ESTs was observed for MeJA treated plants.

Focusing on defense-related processes a well as the jasmonic acid (JA), ethylene (ET)- and salicylic acid (SA) pathways, five further categories were selected and three of them revealed R statistic values >3 for at least one pair-wise comparison of EST abundances by treatment (Figure 2b). For egg-induced plants (EF), the GO analysis indicated a particular increase in the proportion and variety of expressed genes involved in the “defense responses” and the “responses to jasmonic acid / ethylene dependent systemic resistance”. In both cases class I chitinases (Unitrans: elm_00100, data not shown) appeared to be responsible for much of the observed differential expression. Lipoxygenases appeared to be responsible for differential expression in the category “response to JA stimulus”, which is consistent with the result in the category “fatty acid biosynthesis”. On the other hand, GO analysis indicated no significant differences between the compared treatments in transcript abundances involved in transport, carbohydrate metabolism, signal transduction, translation, transcription, ET- and SA-pathways (Figure 2a and b).

**Figure 3:**
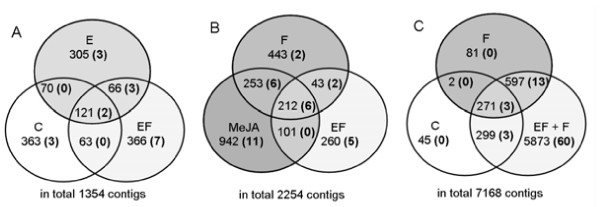
**Comparison of Unitrans abundances (E value thresholds ≤1e-20) for genes differentially expressed in*Ulmus minor*plants subjected to various treatments:A)***Xanthogaleruca luteola* feeding together with egg laying (EF), artificial scratching with transferred egg clutches (E), untreated control (C); **B)***X. luteola* feeding (F), methyl jasmonate (MeJA) and EF; **C)** mixed library (EF + F), C and F; genes in brackets were classified in terms of the Gene Ontology (GO) category “defense response” (E-value ≤ 1e-20). The Unitrans belonging to each category are listed in Additional files 6, 7, and 8.

The distribution of Unitrans ≥ 2 ESTs between the different treatments annotated against the plant taxonomic UniProt database is shown in the Venn diagrams of Figure 3. Focusing on the analysis of the “egg”-induced treatment (E) and the mixed library EF + F, the pairwise intersections between the C, E and EF treatments are about 30% of the Unitrans (Figure 3A). When including data from the other treatments, half of the Unitrans for the EF or F treatments overlap with MeJA (Figure 3B). Interestingly around 90% of the C and F treatment Unitrans overlap with the those from the (10–17 fold larger) mixed sample EF + F (Figure 3C). This suggests that many of the assignments that are apparently unique to one treatment may well be shared with other treatments, but insufficient sequence coverage prevented detection in these other samples. We have highlighted (in parentheses) those transcripts assigned to the gene ontology category “defense response” in the Venn diagrams (Figure 3, A–C). As expected, only a small number of Unitrans from the untreated plants (C) were found to be assigned to this category. All Unitrans related to defense were detected in treatments that include induction by eggs (E, EF and EF + F). Here the Unitrans number increased with the library size. Table 2 shows a list of Unitrans with predicted gene functions belonging to the GO category “defense response”.

**Table 2:** Relative abundance of Unitrans annotated as having a predicted function in defense response in six libraries representing different elm leaf treatments

**Gene description based on homology**	**# of Unitrans**	**# of ESTs**	**Best database match**	**E-value**	**Treatment (pptt)**					
					**EF**	**EF + F**	**E**	**F**	**MeJA**	**C**
**PR Proteins**										
Class I chitinase	12	2111	*Brassica napus*	8e-58	**161**	70	28	13	133	33
Disease resistance response protein	5	192	*Arabidopsis thaliana*	4e-25	-	1	-	-	1	-
Glucan endo-1,3-beta-glucosidase	7	190	*Prunus persica*	2e-115	10	2	**19**	-	7	-
Cysteine proteinase inhibitor	3	189	*Vigna unguiculata*	1e-26	5	5	-	-	6	5
MLP-like protein	3	86	*Arabidopsis thaliana*	3e-42	**86**	10	2	3	8	3
Pathogenesis-related protein	3	34	*Medicago truncatula*	5e-22	-	0.3	-	2	-	-
MLO-like protein	2	4	*Arabidopsis thaliana*	4e-32	-	0.1	-	-	-	-
**Phytohormone signaling**										
Jasmonate ZIM-domain protein 3	1	111	*Arabidopsis thaliana*	3e-24	**21**	3	-	2	10	-
Ethylene-responsive transcription factor	3	97	*Arabidopsis thaliana*	1e-41	-	3	5	2	4	9
Auxin signaling F-box 2	2	33	*Arabidopsis thaliana*	3e-79	**21**	1	5	2	1	-
ABC transporter G family member 40-	1	10	*Arabidopsis thaliana*	1e-62	-	0.1	-	0.1	1	-
Regulatory protein NPR1	1	7	*Arabidopsis thaliana*	1e-25	**9**	-	0.2	0.3	-	-
Coronatine-insensitive protein 1	1	8	*Arabidopsis thaliana*	9e-54	-	0.1	-	-	-	-
Probable WRKY transcription factor 33	1	4	*Arabidopsis thaliana*	7e-28	16	-	-	-	-	-
Ethylene-insensitive protein 2	1	2	*Arabidopsis thaliana*	9e-23	-	0.1	-	-	-	-
**Jasmonic acid synthesis**										
Allene oxide synthase	4	391	*Linum usitatissimum*	3e-39	10	12	9	13	30	5
Peroxisomal acyl-coenzyme A oxidase 1	2	8	*Arabidopsis thaliana*	6e-35	**5**	0.1	-	-	-	-
Innate immunity										
Pre-mRNA-splicing factor SPF27	1	13	*Arabidopsis thaliana*	3e-52	-	0.5	-	-	-	-
Pre-mRNA-processing factor 19	4	11	*Arabidopsis thaliana*	2e-29	-	0.3	-	-	1	-
Cell division cycle 5-like protein	2	11	*Arabidopsis thaliana*	5e-50	-	0.3	-	-	-	-
Protein pleiotropic regulatory locus 1	2	7	*Arabidopsis thaliana*	5e-75	-	0.3	-	-	-	-
Serine / threonine-protein kinase PBS1	1	2	*Arabidopsis thaliana*	7e-42	-	0.1	-	-	-	-
**Regulatory role in defense response**										
Patatin-like protein	1	557	*Solanum tuberosum*	7e-83	**47**	13	9	15	48	-
Heat shock protein 81	4	309	*Arabidopsis thaliana*	0	-	**5**	-	2	3	-
Ankyrin repeat domain-containing protein 2	4	136	*Arabidopsis thaliana*	5e-59	-	3	9	19	4	-
(+)-neomenthol dehydrogenase	3	29	*Arabidopsis thaliana*	2e-23	-	1	-	2	1	-
Cyclic nucleotide-gated ion channel	3	15	*Arabidopsis thaliana*	2e-45	**10**	0.3	-	0.1	-	-
Two pore calcium channel protein 1	2	5	*Nicotiana tabacum*	6e-31	-	0.2	-	-	-	-
**Cell wall metabolism**										
Cellulose synthase A catalytic subunit 3	3	17	*Arabidopsis thaliana*	5e-63	-	1	-	-	-	-

Libraries: C (untreated control), E (artificial scratching & eggs transferred), EF (egg laying & feeding), F (feeding), MeJA (methyl jasmonate), mixed library EF + F. Relative Unitrans abundance calculated on library counts by parts per ten thousand (pptt) based on the annotation to Plant Swiss Prot (BLASTx, E-value ≤ 1e-20). Annotated transcripts filtered depending on their predicted function to the category “GO:0006952 defense response” of the Gene Ontology term. **Bold** = increased relative Unitrans abundance in egg-induced plants.

**Figure 4:**
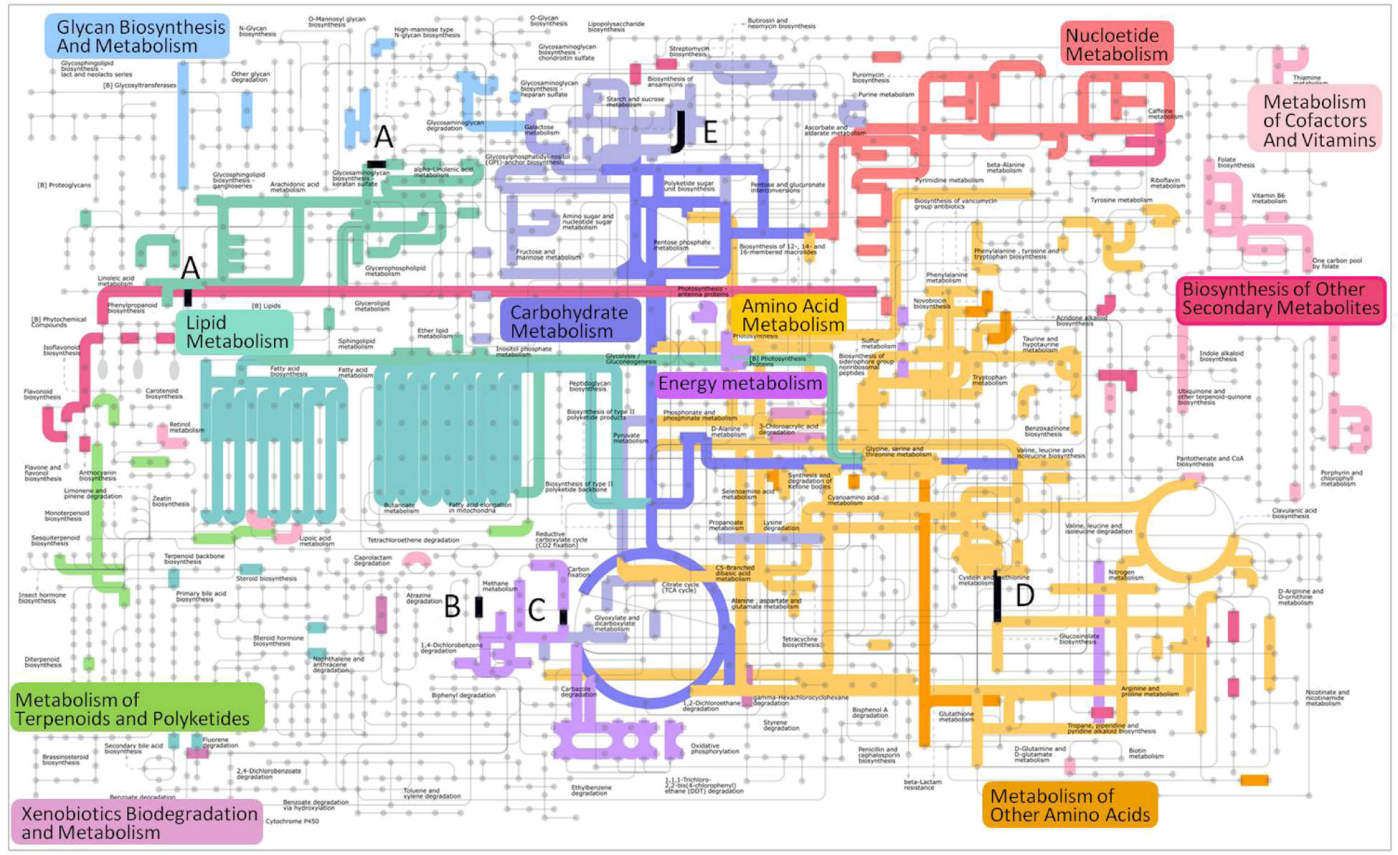
**Metabolic pathways expressed in*Ulmus minor*after egg laying and feeding by the elm leaf beetle*Xanthogaleruca luteola*.** 451 enzymes (based on EC numbers, shown as bold colored lines) identified *via* Blast searches against the UniProt database (E-value ≤1e-40) were used to generate the map with iPath [43], a web-based tool for the visualization of metabolic pathways. Enzymes A–E highlighted in **black** are referentially expressed in egg-induced plants (see Results).

For visualization of metabolic pathways represented by gene transcripts, maps were reconstructed with the iPath software [43], using enzymes corresponding to the annotated Unitrans. The enzymes are designated by the usual enzyme commission (EC) nomenclature. Cross-comparisons among treatments (EF, F, C, E, and MeJA) demonstrate that most enzymes are only expressed in one of the two compared treatments below (Additional file 9). Because library size had a strong influence on the extent of the annotated and mapped enzymes, we mapped the largest library, EF + F, in which most transcripts of the other libraries occur (for data on F and C libraries see Venn diagram in Figure 3C and for MeJA, EF libraries data not shown). We used the 451 EC numbers of the EF + F library to generate a metabolic map to examine putative biochemical pathways present in feeding- and egg-induced *U. minor*, and also highlighted those putative enzymes preferentially expressed in egg-induced plants (Figure 4). Enzymes associated with primary metabolism (carbohydrate-, amino acid-, nucleotide-, energy- and lipid metabolism) are predominant, whereas enzymes associated with secondary metabolism (e.g. phenylpropanoid, flavonoid, and terpenoid biosyntheses) are much less prevalent.

To elucidate the molecular basis for the biosynthesis of volatiles involved in indirect defenses of elm to leaf beetles, we mainly focused on terpenoid metabolism comparing the different treatments with iPath, a web-based tool for the visualization of metabolic pathways. According to the different iPath maps, the enzymes involved in terpenoid biosynthesis were most frequently observed in the large treatment combination EF + F (Figure 4, Additional file 9). Several transcripts involved in terpenoid biosynthesis including prenyltransferases and terpene synthases were found, but low EST numbers made a statistical analysis between treatments impossible (data not shown). Putative enzymes with increased transcript abundances in the EF versus MeJA, F, E, and C treatments with significant Rstat values (highlighted in the map) are lipoxygenase (A = EC:1.13.11.12; oxylipin [octadecanoid] metabolism), catalase (B = EC:1.11.1.6; hydrogen peroxide catabolic process), glyceraldehyde-3-phosphate dehydrogenase (C = EC:1.2.1.13; glycolysis), cobalamin-independent methionine synthase (D = EC:2.1.1.14; methionine metabolism), and sucrose synthase (E = EC:2.4.1.13; sucrose metabolism). The EC numbers used for generating maps are listed in Additional file 10, showing the normalized counts for Unitrans and R values for the different cross-comparisons between treatments.

The Unitrans associated with the GO category “defense response” included genes for pathogen related proteins (PR), phytohormone signaling, plant innate immunity, and other regulatory processes (Table 2). Cross-comparison of the different treatments revealed genes with increased transcript abundances in egg- and feeding-treated plants. Ten putative genes were specifically enhanced in all the insect egg-treatments (libraries EF, E and EF + F) in comparison to the other treatments. These were annotated as: a class I chitinase, a glucan endo-1,3-beta-glucosidase, a MLP-like protein, a jasmonate ZIM-domain protein, an auxin signaling F-box protein, the regulatory protein NPR1, a peroxisomal acyl-coenzyme A oxidase, a patatin-like protein, heat shock protein 81, and a cyclic nucleotide-gated ion channel) (bold numbers Table 2). The most abundant transcripts in this group were the class I chitinase (2111 ESTs), the heat shock protein 81 (309 ESTs), and the glucan endo-1,3-beta-glucosidase (190 ESTs). Interestingly five of these transcripts showed simultaneous increases in the MeJA–treated plants, again suggesting a role for MeJA in response to egg laying. Ten putative genes were present at low transcript abundances (2-17 ESTs) exclusively in those plants that were induced by egg laying, and almost all of these were from the large EF + F library. These were annotated as: MLO-like protein 6, coronatine-insensitive protein, WRKY transcription factor 33, ethylene-insensitive protein, pre-mRNA-splicing factor, cell division cycle 5-like protein, protein pleiotropic regulatory locus, a serine / threonine-protein kinase, two pore calcium channel proteins, and cellulose synthase A catalytic subunit 3. Three genes (ABC transporter, allene oxide synthase, and pre-mRNA-processing factor) showed apparent increases in MeJA induced plants (10-391 ESTs). Two additional gene transcripts (pathogenesis-related protein, and ankyrin repeat domain-containing protein) showed increased abundance in feeding-induced plants (34-136 ESTs). Transcripts annotated as an ethylene-responsive transcription factor were enhanced in untreated plants (97 ESTs).

From the 15 most abundant protein transcripts in egg- and feeding-treated plants, the three with EST counts >1000 were (a) lipoxygenase which is involved in JA biosynthesis, (b) a sieve element-occluding protein preventing the loss of photoassimilates after wounding [44] and (c) catalases which are known to serve as common antioxidant enzymes and to induce suberization and other protective mechanisms after wounding [45] (Table 3). Four proteins with EST counts >100 were (d) peptidyl-prolyl cis-trans isomerases which are also known as cyclophilins and accelerate the folding of proteins [46], (e) proteasome subunits responsible for protein degradation and turnover [47], (f) auxin-repressed proteins known to affect auxin signaling as negative regulators [48] and (g) methionine synthase (cobalamin-independent), which catalyses the last step in the production of the amino acid L-methionine used by plants for many essential direct or indirect cellular processes [49]. Two further proteins almost unique to the EF library in these elms were (h) the enzyme methionine sulfoxide reductase, which functions in plant defense *via* the regulation of the cell redox status and is known to be involved as an antioxidant in repairing proteins damaged by oxidative stress [50], and the transport protein SFT2, which in yeast is involved in traffic to the Golgi complex and vesicle-associated membrane fusion [51,52]. The R statistic was applied in order to detect differences in relative transcript abundances between the elm treatments [42]. Transcripts with R > 3 (~99% true positive rate for our libraries) were considered to be differentially expressed between the libraries. For all these protein types, the R statistic revealed a significant difference in transcript abundances between the treatments.

**Table 3:** **Identification of putative regulatory proteins with high occurrence in egg laying- and feeding-induced*Ulmus minor*leaves**

**Pfam accession**	**Gene description**	**# of ESTs**	**GO Biological process**	**Treatment (pptt)**						
				**EF**	**EF+**	**E**	**F**	**MeJA**	**C**	**R**
PF00305	Lipoxygenase	1602	lipid biosynthetic process	110	43	33	38	162	38	30.9
No family	Sieve element-occluding protein	1545	-	245	40	66	19	86	33	27
PF00199	Catalase	1159	response to stress	73	24	28	25	54	19	6.7
PF00160	Peptidyl-prolyl cis-trans isomerase	773	protein folding	52	15	9	19	34	33	5.3
PF00227	Proteasome subunit	341	response to stress	21	8	5	4	24	9	3.9
PF05564	Auxin-repressed protein	207	signal transduction	26	3	-	2	-	-	4.3
PF01717	Methionine synthase	200	methionine biosynthetic process	42	4	-	2	16	9	8.7
PF01641	Methionine sulfoxide reductase	58	catalytic activity	34	1	-	-	-	5	8.3
No family	Protein transport protein SFT2	16	vesicle-mediated transport	78	-	-	-	-	-	29.9

Treatments: C (untreated control), E (artificial scratching & eggs transferred), EF (egg deposition & feeding), F (feeding), MeJA (methyl jasmonate), mixed library EF + F. Relative Unitrans abundance calculated on counts by parts per ten thousand (pptt) based on the annotation to Plant UniProt (BLASTx, E-value ≤1e-20). Transcripts correlated on their predicted function to the Pfam = protein family database. R–values >3 were considered as significantly differentially expressed for the respective treatment against C (true positive rate of ~99%) by Test Statistics R [42].

## Discussion

The large-scale EST sequencing results shown here represent the first step in studying the defensive responses of field elms to egg laying by the specialist elm leaf beetle *Xanthogaleruca luteola*, at a molecular level. 361,196 expressed sequence tags (ESTs) were assembled into 52,823 unique transcripts (Unitrans). Although the gene discovery rate among the transcripts was low due to the low number of *Ulmus* genes in public databases, we were nevertheless able to identify a large number of candidate genes with possible roles in the response of elm to egg laying by the elm leaf beetle. Normalization based on sequence sample size and analysis using R statistics provided the basis for comparative gene expression analysis using EST frequencies across five different biological treatments: egg laying and feeding by *X. luteola* (EF), feeding (F), transfer of egg clutches (E), methyl jasmonate spraying (MeJA) and an untreated control (C). The function of these candidate genes must now be confirmed in further studies. Despite a similar sample size and the fact that clonal plant material, identical sequencing technologies, and sequence assembly were used, the EST frequencies of the five treatments showed astonishingly small intersections as can be seen in the Venn diagrams and visualization of metabolic pathways (Figures 3 and 4). Therefore, although the influence of *X. luteola* feeding on transcripts cannot be ruled out, the ten-fold larger library EF + F is still capable of being used for detecting the less abundant transcripts induced by egg laying, as it represents a broad snapshot of the transcriptome and of the activity in the different biochemical pathways in elm. We compared Unitrans distributions and gene ontology (GO) terms and identified enzyme differences among the treatments especially with regard to egg-induced changes in transcript abundances.

### Leaf beetle egg laying increases defense gene transcripts and decreases transcripts for photosynthesis

Gene ontology analysis indicated a decrease in the transcription level for those genes involved in photosynthesis in the egg- and MeJA-induced plants. Egg laying by herbivorous insects can cause a reduction in photosynthetic activity, as has been shown for a tree species (*Pinus sylvestris*) and a crop plant ( *Brassica oleracea* L.) [53,54]. Whether transcription of photosynthesis genes in egg-free leaf parts is affected by eggs has not been studied so far. There has been only one previous study showing a reduction of transcription of photosynthesis-related genes after egg laying; however, in this study tissue situated directly underneath the egg masses without full access to light had been sampled [31]. In our study, the material sampled for sequencing included leaf tissue immediately adjacent to the egg laying site as well as that some distance away. The analyzed tissue was not covered by eggs and had full access to light, and thus the response seen in photosynthesis-related genes is not just a response to low light. Our results are consistent with that of other studies showing the reduction of photosynthesis-related genes after MeJA treatment [55,56].

Further it appears that MeJA affected transcript levels in a manner similar to the insect treatments, which has also been observed in several other studies of plant responses to insect feeding damage [57-60]. The transcripts of MeJA treated plants showed GO term distributions similar to the transcripts of EF treated plants. Both egg laying (represented by the two libraries EF and EF + F) and JA (or MeJA) treatments induce the indirect defenses of elms by stimulating the emission of volatiles that attract egg parasitoids. Nevertheless, these different experimental treatments induce volatile patterns that differ qualitatively and quantitatively ([8,39], Meiners T. unpublished data). In contrast, only minor differences in the overall transcript levels were detected between untreated plants and plants with transferred eggs, indicating that the experimental imitation of the egg laying event does not cause any wholesale change in transcriptional levels.

The GO analysis indicated an increase in the number and quantity of expressed genes involved in defense responses for egg-induced plants. In a similar way, an inverse correlation between photosynthesis- and defense-related genes was observed in *Arabidopsis thaliana* after egg laying by *Pieris brassicae*[31], which might indicate a reallocation of resources from primary to secondary metabolism. However, in *Brassica oleracea* var. *gemmifera,* only a few defense genes were found to respond to treatment of leaves with pierid eggs [32].

### Induced defense genes encode PR proteins, chitinases, WRKY transcription factors and other proteins

In this study, special attention was paid to the detection of expressed genes associated with plant defense against insect eggs, as indicated by enhanced transcript abundances after egg laying in comparison to the other treatments. In egg-induced plants, we observed an increase in transcripts annotated as chitinases, glucan endo-1,3-ß-glucosidases, pathogenesis-related protein (PR), major latex protein (MLP), heat shock protein 81, patatin-like protein, NPR1, and WRKY transcription factor 33. In *Ulmus americana* similar upregulation of chitinase and PR-1 transcripts were induced after inoculation with the fungus *Ophiostoma novo-ulmi* at a similar time point (48–72 h) after treatment [30]. Almost all of the 53 upregulated transcripts reported in this study with sequence similarities to defense related proteins were also found in our much larger *U. minor* database. PR proteins are well known to be involved in defense responses after herbivore attack [61]. Our results suggest the potential importance of *de novo* PR protein expression by *U. minor* in response to attack by *X. luteola*. Transcripts detected with high expression in egg-treated elms show sequence similarities to genes belonging to different PR protein families (PR-1, PR-2, PR-3, and PR-10). Chitinases (PR-2) play a direct role in plant defense by degrading microbial cell wall components, often coordinated with the induction of glucan endo-1,3-ß-glucosidases (PR-3), and seem to be a prominent feature of the inducible defense profile after pathogen attack [4,30,62]. Our data suggest that this is also true after insect attack in trees. Chitinases and glucan endo-1,3-ß-glucosidase are also known to be induced at and near the egg laying site in *A. thaliana* by pierid eggs and could play a defensive role against newly hatched larvae [31]. Chitin is an important structural component of the exoskeleton and the midgut in all insects [63,64]. Chitinases might also be effective defenses against the egg stage even though chitin-like components are not known from egg shells except in mosquitoes [65]. But, if chitinases were to penetrate the eggs they could prevent larvae from hatching, and might serve as a direct defense against the beetle eggs.

MLP-like proteins belong to the PR-10 protein family, which are induced by both biotic and abiotic stress conditions in various plant tissues [61]. The biological function of these proteins remains to be elucidated, but they very likely participate in binding of ligands, such as plant hormones and secondary metabolites [66]. Many PR genes are regulated by WRKY transcription factors, and WRKYs are known to fine-tune stress responses, including defense responses [67]. WRKY 33 initiates the positive regulation of JA-induced defense genes and negative regulation of SA-related defense genes [68]. WRKY factors allow binding to the W-box motif, which is found in promoters of PR defense genes such as PR-10 [69] and chitinase [70]. W-boxes have also been identified in the promoter region of *NPR1,* an important receptor which helps to regulate SA/ JA-phytohormone signaling [71].

Two proteins which also showed increased expression in egg-induced elms are patatin-like protein and heat shock protein (HSP) 81. Patatin proteins are related to the major storage protein known from potato tubers and have the enzymatic activity of phospholipases and release fatty acids from membrane lipids. These proteins have been identified in many plant species and were shown to be involved *inter alia* in pathogen-triggered cell death and to be induced by wound stimuli [72]. They might also be associated with the herbivore-induced defense pathway *via* the mobilization of linolenic acid from the cell membrane, which activates the octadecanoid pathway and finally leads to the synthesis of JA and other oxylipins [73,74]. HSPs meanwhile, are molecular chaperones which can modulate the folding of a variety of other specific target proteins involved, for instance, in cell cycle control and signal transduction [75]. HSP 81 belongs to the HSP 90 family of stress proteins, which are known to influence several resistance-gene signaling pathways, the inhibition of which lead to decreased resistance to pathogens and increased resistance to insect herbivores [76,77]. Thus, a suite of defense response genes, that work together to protect the plant from insect attack appears to be coordinately activated by egg laying on elm.

### Transcripts of jasmonic acid biosynthesis genes are present in high abundance

JA has been determined to be an integral part of the plant signal transduction pathway, which leads to the activation of direct- and indirect defenses against herbivorous insects [36,78,79]. Decreased resistance to herbivores and enhanced egg laying activity has been observed in tomato mutants with impaired JA biosynthesis [80]. Moreover, transcriptome analyses using microarrays indicated that a large portion of herbivory-induced responses are mediated through the JA pathway [58,81].

In egg-induced elms, we found high levels of transcripts of genes encoding key enzymes involved in the biosynthesis of JA including lipoxygenase and allene oxide synthase. Our findings support the expected involvement of the octadecanoid signal transduction pathway in egg-induced plant defense, as the treatment of elms with MeJA leads to the release of volatiles that are attractive to egg parasitoids. Genes involved in JA biosynthesis were also upregulated after pierid eggs laying on *A. thaliana*[31]. However, we also found enhanced transcript abundances after egg laying in comparison to the other treatments for jasmonate ZIM-domain proteins, which are known to repress JA responsive genes [38]. Auxin might be another phytohormone involved in elm responses to eggs, and transcripts of both positive and negative regulators of auxin signal transduction, an auxin receptor (Auxin signaling F-box 2) and an auxin-repressed protein, were also found [48,82]. After JA treatment of poplar, down regulation of genes involved in auxin signaling was observed [83]. Auxin interferes with JA and SA signaling, and the negative regulation of auxin is supposed to mediate adaptive response to biotic stress [84,85]. Another hormone, salicylic acid, may also be involved in plant responses to eggs since SA-deficient mutants of *A. thaliana* showed different responses to pierid eggs than wild type plants [86]. Further studies are necessary to understand the role of JA in concert with other phytohormones in signaling in order to regulate egg-induced defenses.

### Gene transcripts for terpenoid biosynthesis were detected at only low levels

There is strong evidence that damage-dependent JA levels activate distinct sets of defense genes leading to terpenoid formation [87]. To elucidate the molecular basis underlying volatile biosynthesis associated with the indirect defenses of elm in response to egg laying, we compared the different treatments with reference to transcripts involved in terpenoid metabolism. Although it has been established previously that a volatile blend with an enhanced fraction of terpenoids that is attractive to egg parasitoids is produced by these elms 2–3 d after egg laying [22], we detected only a few transcripts involved in terpenoid metabolism in the elm leaves following egg treatment. The respective genes may be differentially expressed, but below the detection threshold of our analysis or else possibly the expression is not controlled at the transcript level. In general it is supposed that herbivore-induced *de novo* production of terpenoids takes place several hours following the activation of terpene synthase genes [87]. Enhanced abundance of transcripts for terpene synthases were also found in samples taken from the needles of *Pinus sylvestris*, that were laden with eggs of the herbivorous sawfly *Diprion pini*; these egg-laden pine needles emit a volatile terpenoid blend that attracts egg parasitoids. However, transcript levels for a sesquiterpene synthase from *P. sylvestris* which produces ( *E*)- *β*-farnesene, the compound responsible for the attraction of an egg parasitoid of sawfly eggs, were not enhanced by *D. pini* egg laying [41].

The time window in which egg-induced elm leaf material was harvested for sequencing and the large size of our database should have enabled the detection of even relatively rare transcripts associated with the early and late direct and indirect defense responses against the leaf beetle. In *A. thaliana* the number of up- or down-regulated genes increased as time elapsed from 1–3 d after pierid eggs have been laid on plants [31]. Because transcripts for terpenoid metabolism are under-represented in our database, we can only speculate about the molecular basis of egg-induced volatile production for indirect defense in elm. We hypothesize that egg-enhanced JA levels increase transcript abundances for JA biosynthesis genes, thereby activating so far unidentified genes which stimulate the emission of a volatile blend of terpenoids from elms, but by a mechanism that does not involve an increase in the transcript levels for the genes associated with the formation of these compounds, as has been demonstrated for other plants [41,88,89].

Since plant defense signaling mechanisms may well be selected to respond as rapidly as possible to the presence of herbivores, their initial response is probably modulated by physiological means in the first instance, rather than by changes in expression levels. To confirm this hypothesis further studies are needed to measure the levels and activities of terpenoid biosynthetic enzymes participating in volatile formation.

### Transcripts were induced encoding other protein types

In addition to transcripts for proteins known to be involved in defense responses, we found enhanced transcript abundances of proteins (and protein families) in egg-induced plants for which little knowledge is available on their possible role in defense responses towards insect eggs. These proteins are assigned to general functions, such as stress response, protein metabolism, signaling and transport. They probably represent a critical link between defense and developmental processes in these plants. Next to the up-regulation of lipoxygenase especially high EST numbers and a strong significant difference between the treatments were found for transcripts associated with sieve element-occluding proteins, which supposedly play a role under stress conditions after insect attack [90]. Among the enhanced transcript abundances in egg-induced plants high EST numbers were found for transcripts of catalases, which protect cells from the toxic effects of reactive oxygen species (ROS) such as hydrogen peroxide, which are often found in stressed tissues [45]. Herbivory has been found to elicit the production of ROS that are involved in further downstream transduction cascades, leading to the induction of defense-response genes [35], as well as in localized cell death [91]. We hypothesize that enhanced ROS levels caused by injury during egg laying are most likely responsible for the increased expression of related classes of catalases in elm, where localized cell death has been observed under the egg clutches [13].

Interestingly high EST numbers of trancripts associated with methionine metabolism were found in egg-induced plants. An increase of methionine synthase after MeJA treatment was also reported for *A. thaliana*[55]. The proteinogenic amino acid L-methionine has many essential direct and indirect functions in cellular metabolism, including ethylene biosynthesis [49], as well as the biosynthesis of defense compounds [92]. High EST numbers were also found for transcripts involved in protein folding (cyclophilins) and degradation (proteasome subunits), possibly indicating that turning over and re-configuring the proteome might be a critical step in the defensive responses of plants, as well possibly having an important role in signal transduction [93], including the fine-tuning of JA signaling [94]. Among those gene trancripts that were enhanced by elm beetle egg laying, we also identified transcripts associated with proteins involved in the transport of ions and other compounds, such as cyclic nucleotide-gated ion channels [95], and the transport protein SFT2, albeit with lower EST number. Especially interesting among these is the transport protein SFT2, as this was exclusively present in leaf samples after egg laying treatment. SFT2 is a member of the SNARE protein family, which is known to function in vesicle-associated membrane fusion events during transport processes in plants. Plant SNARE proteins are thought to be involved in developmental processes and pathogen defense, but it remains unproven whether SFT2 functions like their yeast counterpart [52,96].

## Conclusions

While insect feeding is known to trigger major changes of the transcriptome in herbaceous and woody plants (e.g. [58,83,97,98]), insect egg laying has so far only been shown to elicit large scale changes in the transcriptome of herbaceous plants [31,32]. Our elm EST database shows for the first time that insect eggs can induce similarly transcriptional changes in a woody plant, a deciduous tree. There was a pronounced shift towards transcripts involved in general stress responses such as oxidative stress (catalases, methionine sulfoxide reductase), and defense responses (PR proteins), phytohormone signaling (in particular JA), and transport processes (cyclic nucleotide-gated ion channels and transport protein SFT2). Further changes were observed in primary metabolism (sucrose synthase, glyceraldehyde-3-phosphate dehydrogenase, methionine synthase, and cyclophilins), and a possible downregulation of photosynthesis suggests a metabolic shift from growth and development to defense. As such, this work presents a large data set from a well established, ecological natural plant – insect system which will be important for further studies of the mechanisms of direct and indirect plant defenses against insects and other serious pests such as the Dutch elm disease fungi.

## Methods

### Plants

All plants originated by propagating a single genotype of the European field elm, *U. campestris*, referred to as *U. campestris* cv. ‘Dahlem’, that originated from a forest 50 km east of Berlin, Germany. Shoots were maintained by monthly subculture on DKW propagation medium, which contained 1 mg dm^-3^ 6-benzylaminopurine (BAP; Sigma) and 0.01 mg dm^-3^ indole-3-butyric acid (IBA; Sigma) [99,100]. Rooted shoots were produced by transferring 3–5 cm shoots from the propagation medium (above) on DKW media containing 3 mg dm^-3^ IBA hormone and no BAP. After 3–5 days shoots were transferred into soil and grown in a climate chamber (22°C, 55% relative humidity (RH), 150–200 μmol m^-2^ s^–1^ PAR) under a 16 h /8 h light:dark (LD) photoperiod. To rear mature plants, shoots were transferred individually in plastic pots (11 × 11 × 12 cm) filled with potting soil (type T, Kausek GmbH, Germany). All experiments were conducted with 3–4-month-old elm plants with 15–20 leaves and a height of about 50 cm. Elms generated from this culture were found to retain their responses to the beetles [22].

### Insects

Adults of *Xanthogaleruca luteola* (Coleoptera: Chrysomelidae) were collected in the environs of Montpellier and Perpignan (France) and in Palava (Spain). Adult beetles and hatching larvae were reared in the laboratory in cages (40 × 40 × 70 cm) on ‘Dahlem’ elm plants in the greenhouse (20–40°C, 40–50% RH, 150 μmol m^-2^ s^-1^ PAR) under a 16 / 8 h LD photoperiod. Pupae were transferred in transparent plastic boxes (20 × 20 × 6 cm) for hatching in the climate chamber (see above).

### Treatments

Elm leaf samples were taken at three time points (3 h, 48 h and 72 h) after applying five different treatments (see below) since elms are known to respond to elm leaf beetle infestation by releasing synomones attractive to egg parasitoids in this time scale [21,40]. For each time point and treatment, six replicate plants were harvested. For induction with *X. luteola*, 7–15 beetles were kept within micro perforate plastic bags (180 × 350 mm, Weber Packaging GmbH, Germany) on each treated elm plant. *Egg laying & feeding:* Female beetles were allowed to lay eggs and to feed (leaf material sampled at 48 h and 72 h after egg deposition). *Feeding*: Male beetles were used for feeding experiments (sampling at all time points), in order to exclude any possibility of egg laying in these samples. *Artificial scratching & eggs transferred:* To experimentally mimic the egg laying event by the beetle, leaves were scratched with a scalpel (thus mimicking removal of leaf epidermis by female beetles prior to egg deposition), and eggs were glued with oviduct secretion (which attaches the eggs to the leaves) to the wound (sampled at all time points). *Untreated control:* Intact elm plants with micro perforate plastic bags (sampled at all time points). *Methyl jasmonate:* Elm plants with undamaged leaves were sprayed with 50 ml each plant of an aqueous solution of methyl jasmonate (1 μmol / ml; Sigma, Germany; 95% pure) with 0.05% Tween 20 (for adhesion on leaves) to simulate insect attack (sampled at 24 h). To reduce contaminations by insect material all visible contaminations (eggs and feces) from the insects were removed thoroughly from the leaves with a fine brush.

### RNA isolation and quality control

For isolation of total RNA, elm leaves were removed from stems of variously treated plants, flash frozen in liquid nitrogen and stored at -80°C. RNA was extracted by using a modified method developed for polysaccharide rich plant tissue [101] that employs repeated steps of phenol: chloroform:isoamyl alcohol (PCI; 25:24:1) extraction, and lithium chloride (LiCl) and ethanol precipitations over night. All glassware was treated with RNase AWAY^®^ (Roth, Germany) and RNAse-free water. Plant material (0.5 g) was mixed with 10 ml lysis buffer (0.2 M Tris-HCL, 0.1 M LiCl, 5 mM Na_2_EDTA adjusted to pH 8.2) to which 1% SDS, 0.01% ß-mercaptoethanol, 9% sodium acetate (2 M, pH 4) 10 ml phenol, 2 ml chloroform and 2% polyvinylpolypyrrolidone (PVPP) were added. The tubes were shaken (15 min, 250 rpm), then centrifuged (15,557 ×g, 4°C, 20 min), and the RNA was extracted three times with PCI. RNA was precipitated with LiCl (2 M final concentration) and collected in high speed 30 ml KIMBLE glass tubes (Kimble, Glass Inc., Vineland, NJ, USA) by centrifugation at 15,557 ×g for 60 min and finally precipitated with three volumes ethanol and 1/10 vol sodium acetate (3 M, pH 5.8) in 1.5 ml plastic tubes. For final purification and removal of genomic DNA, the RNeasy plant mini kit (Qiagen, Germany) including the on-column DNaseI treatment step was used. Aliquots of each purified RNA extract sample were prepared, and RNA concentration was determined spectrophotometrically at 280 and 260 nm. For final quality control and quantification, the total RNA samples were analyzed with an Agilent 2100 Bioanalyzer and Nano RNA 6000 chips (Agilent Technologies, Palo Alto, CA, USA) using the Expert Software (Agilent, version B.02.02.SI258). Total RNA extract samples were immediately frozen for long term storage as ethanol precipitates at −80°C.

### cDNA library construction and 454 sequencing

For cDNA preparation, total RNA from six plant replicates and different time points of each of the respective treatments was pooled together. cDNA was synthesized using the SMART cDNA library construction kit (Clontech, Mountain View, CA, USA). First-strand cDNA was synthesized for each library from 0.5–1.0 μg of total RNA in a 10-μl reaction as described in the kit protocol using the SMART IV primer (AAGCAGTGGTATCAACGCAGAGTGGCCATTACGGCCGGG), a modified oligo(dT) primer (TAGAGACCGAGGCGGCCGACATGTTTTGTTTTTTTTTCTTTTTTTTTTVN), where V = A, G, or C and N = A, G, C, or T), and SuperScript II reverse transcriptase (Invitrogen, Carlsbad, CA, USA). Double-stranded cDNA was synthesized using the modified oligo(dT) primer and the SMART 5´ PCR primer (AAGCAGTGGTATCAACGCAGAGT) followed by a SfiI digestion as described in the SMART kit protocol. Amplified cDNA was purified using the QIAquick purification kit (Qiagen, Hilden, Germany). All column elutions for a specific library were pooled, and the relative cDNA concentration was estimated by running a 1% agarose gel electrophoresis with ethidium bromide staining and comparison to a standard molecular weight ladder. The first round of sequencing involved the use of equal amounts of all five libraries (EF, F, E, MeJA, and C) and ligating them to the 454 adapters as described in the original 454 paper [102]. The second round involved an individual mix containing 3.0 μg of each of the F and EF libraries. Sequencing was done using the GS 20 sequencer (454 Life Sciences, Branford, CT, USA) at the Michigan State University Research Technology Support Facility.

### Bioinformatics: EST processing, assembling, and annotation

The 454 sequencing reads were processed and trimmed to remove low-quality sequence and primer sequences. The trimmed 361,196 high-quality ESTs were used for assembly by the PAVE (Program for Assembling and Viewing ESTs) software package, which incrementally builds unique transcripts (Unitrans) using Megablast for clustering and CAP3 for assembling ESTs [103]. For annotation, sequences were blasted against the plant taxonomic database of UniProt, the full UniProt database (Swiss-Prot and TrEMBL) [104], and the non-redundant NCBI nucleotide database with an e-value threshold of 1e-20. The GO (gene ontology) trees were built using only UniProt annotations that were the best match for a Unitrans (E-value ≤ 1e-40) where at least 60% of the individual ESTs in the Unitrans also matched that protein with an E-Value ≤ 1e-10.

### *In silico* analysis and comparisons of EST libraries

Cross-comparisons between the different libraries were done on the basis of EC numbers, GO categories, and UniProt identifiers. The library counts were normalized based on the library size and displayed as parts per 10,000 (pptt) and parts per 1,000 (ppt). ESTs used in the library counts were required to match the UniProt ID with an E-Value ≤ 1e-10, while their Unitrans were required to match with ≤ 1e-20. This ensures that UniProt IDs identified with high representation in a library are truly representative (i.e., that they align not just to Unitrans from the library, but to parts of the Unitrans containing reads from the library). Significant differences in relative transcript abundances between the GO categories were determined using Fisher's exact test. The R statistic (a log-likelihood ratio) was applied in order to detect differences in relative transcript abundances between the elm libraries. Thresholds with believability greater than 99% (i.e., false positive rate below 1%) were estimated for each library pair individually, using simulations as described in the original reference [42].

Enzymes (EC numbers) identified via Blast searches against the UniProt database (E-value ≤ 1e-40) over queries on the PAVE system were used to reconstruct pictorially biochemical pathway maps using the iPATH software, which can be accessed at http://pathways.embl.de.

### Database web interface

The PAVE elm assembly is accessible through a web interface. It is possible to query the different elm libraries based on ESTs, Unitrans, UniProt IDs / descriptions [104], Protein Families (Pfam) [105], Enzyme Commission numbers (EC) [106] and Gene Ontology terms (GO) [107] without programming knowledge. BLAST searches [108] allow users to blast any sequence (nucleotide or protein) against the elm database. Individually calculated R values are part of the web database display. For further detailed descriptions see “PAVE Information” on the webpage (http://www.agcol.arizona.edu/pave/elm).

### Sequence submission

The 361,196 EST sequences reported in this paper will be submitted to GenBank’s Short Read Archive (http://trace.ncbi.nlm.nih.gov/Traces/sra/sra.cgi) under accession number SRA045857.

## Competing interests

The authors declare that they have no competing interests.

## Authors’ contributions

TM and TF conceived and designed the experiments in cooperation with KB, JG, and MH. TF and KB generated elm clonal material. KB carried out RNA extractions, plant treatments and provided the RNA samples. cDNA libraries were developed by EMD. DG coordinated sequencing. CS, AD and WN performed sequence alignment, assembling, annotation and database construction. Data were analyzed by KB with assistance of TM and DG. KB drafted the manuscript, and all authors contributed and approved the final manuscript.

## Supplementary Material

Additional file 1 **Figure A1.** Size distribution of the unique transcripts (≥2 EST) (=contigs) derived from *Ulmus minor* assemblies. Click here for file

Additional file 2 **Figure A2.** Number of unique transcripts (≥2 EST) (= contigs) derived from *Ulmus minor* assemblies by EST count. Click here for file

Additional file 3 **Figure A3.** Number of ESTs derived from *Ulmus minor* assemblies sorted by open reading frame length (ORF; complete bases); contigs = unique transcripts (≥2 EST). Click here for file

Additional file 4 **Table A1:** Most abundant gene products in *Ulmus minor* leaf EST database. Click here for file

Additional file 5 **Table A2.** Distribution of annotated *Ulmus minor* unique transcripts according to the plant genus. Click here for file

Additional file 6 Excel file of the library comparisons E vs EF vs C of GO category “defense response”, sorted by R stat. Click here for file

Additional file 7 Excel file of the library comparisons F vs C vs EF +F of GO category “defense response”, sorted by R stat. Click here for file

Additional file 8 Excel file of the library comparisons F vs EF vs MeJA of GO category “defense response”, sorted by R stat. Click here for file

Additional file 9 Ipath maps. Click here for file

Additional file 10 Excel file of the Ipath EC query. Click here for file
