# Supplementary figures and images for: An elm EST database for identifying leaf beetle egg-induced defense genes
Source: BMC Genomics. 2012 Jun 15;13:242. doi: 10.1186/1471-2164-13-242 (PMC3439254; doi:10.1186/1471-2164-13-242)

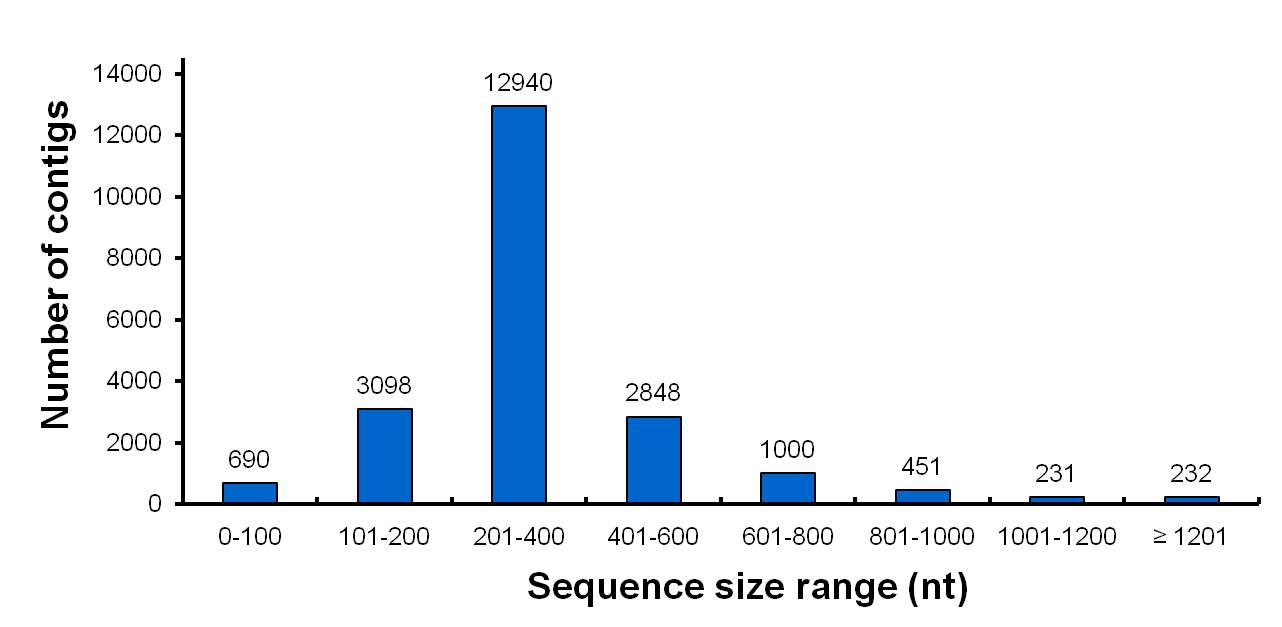


**Figure 1 Size distribution of the unique transcripts derived from *Ulmus minor* assemblies**

Supplement: Additional file 1 — Figure A1. Size distribution of the unique transcripts (≥2 EST) (=contigs) derived from Ulmus minor assemblies. [file 1471-2164-13-242-S1.docx]

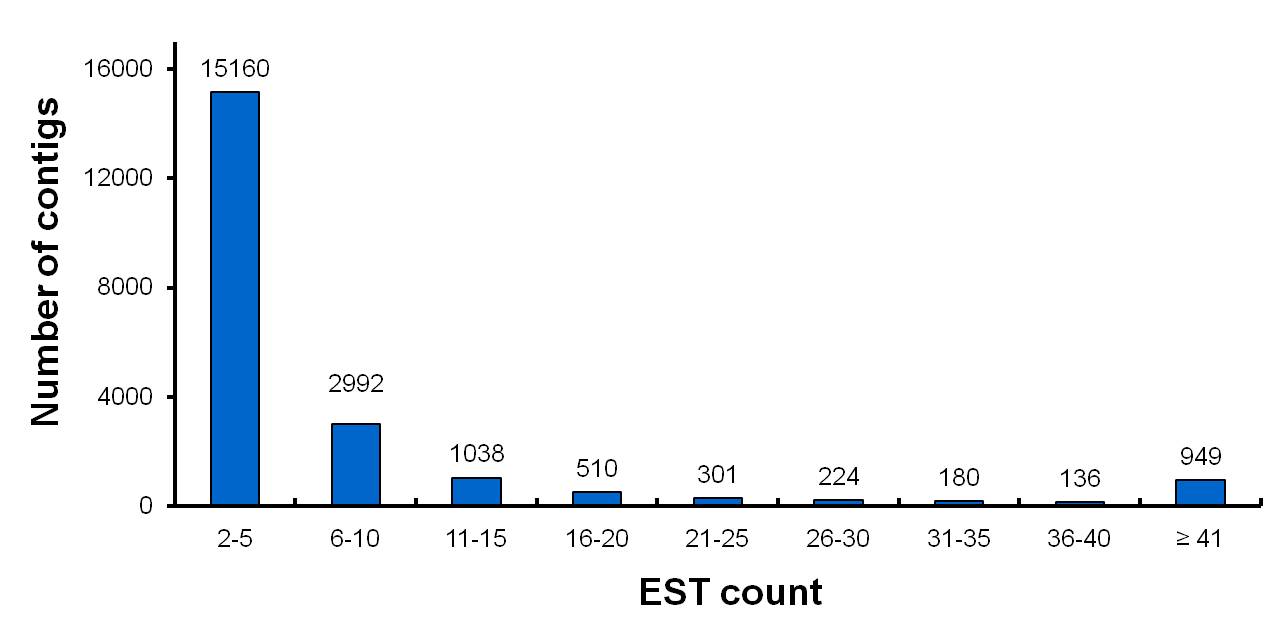


**Figure 2 Number of unique transcripts derived from *Ulmus minor* assemblies by EST count**

Supplement: Additional file 2 — Figure A2. Number of unique transcripts (≥2 EST) (= contigs) derived from Ulmus minor assemblies by EST count. [file 1471-2164-13-242-S2.docx]
