# Supplementary material for: An elm EST database for identifying leaf beetle egg-induced defense genes
Source: BMC Genomics. 2012 Jun 15;13:242. doi: 10.1186/1471-2164-13-242 (PMC3439254; doi:10.1186/1471-2164-13-242)
Supplement: Additional file 3 — Figure A3. Number of ESTs derived from Ulmus minor assemblies sorted by open reading frame length (ORF; complete bases); contigs = unique transcripts (≥2 EST). [file 1471-2164-13-242-S3.docx]

**Table 1 Distribution of annotated** *Ulmus minor* unique transcripts **according to the plant genus**

| **Plant genus** | **Unitrans*** | **Unitrans %** |
| --- | --- | --- |
| *Vitis* (Grape) | 2197 | 25,0 |
| *Ricinus* (Castor bean) | 1825 | 20,8 |
| *Populus* (Black cottonwood) | 1764 | 20,1 |
| [*Arabidopsis*](http://www.uniprot.org/taxonomy/3702) (Mouse-ear cress) | 654 | 7,4 |
| *Glycine* (Soybean) | 522 | 5,9 |
| *Oryza* (Rice) | 148 | 1,7 |
| *Medicago* **(Barrel medic)** | 116 | 1,3 |
| ≤ 1% | 1554 | 17,7 |
| All | 8780 | 100 |

* Unitrans are blasted (BLASTx) against Plant UniProt database,

having a top match with a listed plant genus (E-value ≤1e-20 threshold).
