# Supplementary material for: An elm EST database for identifying leaf beetle egg-induced defense genes
Source: BMC Genomics. 2012 Jun 15;13:242. doi: 10.1186/1471-2164-13-242 (PMC3439254; doi:10.1186/1471-2164-13-242)
Supplement: Additional file 4 — Table A1: Most abundant gene products in Ulmus minor leaf EST database. [file 1471-2164-13-242-S4.docx]

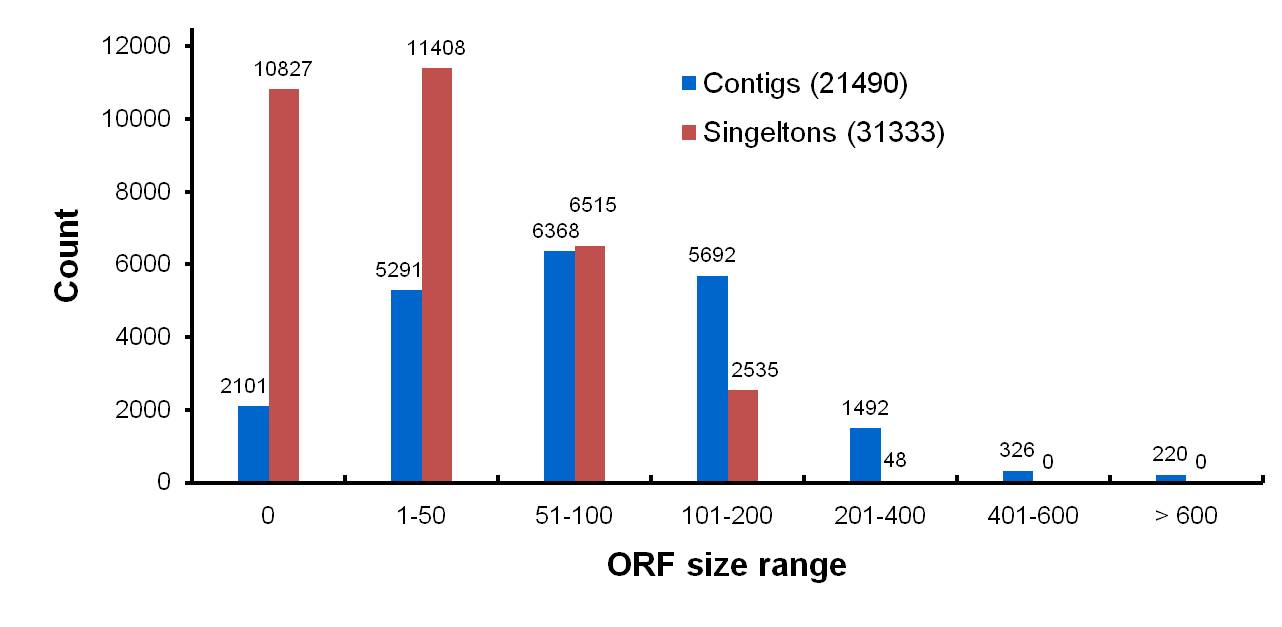


**Figure 3 Number of ESTs derived from *Ulmus minor* assemblies sorted by open reading frame length (ORF; complete bases)**
