# Supplementary material for: An elm EST database for identifying leaf beetle egg-induced defense genes
Source: BMC Genomics. 2012 Jun 15;13:242. doi: 10.1186/1471-2164-13-242 (PMC3439254; doi:10.1186/1471-2164-13-242)
Supplement: Additional file 5 — Table A2. Distribution of annotated Ulmus minor unique transcripts according to the plant genus. [file 1471-2164-13-242-S5.docx]

**Table 2: Most abundant gene products in *Ulmus minor* leaf EST database**

| **Gene description^a^** | **Sum ESTs** | **ESTs %** | **Total Unitrans number** |  | | **Functional classification^b^** | |
| --- | --- | --- | --- | --- | --- | --- | --- |
| Unknown function**^c^** | 168919 | 61,6 | 4798 |  | - | |  |
| Chlorophyll a-b binding protein | 23021 | 8,4 | 40 |  | Photosynthesis | |  |
| Photosystem I+ II protein | 18327 | 6,7 | 43 |  | Photosynthesis | |  |
| Ribosomal protein | 8251 | 3,0 | 197 |  | Translation | |  |
| Retrotransposon protein | 7753 | 2,8 | 2 |  | Unclassified | |  |
| Ribulose bisphosphate carboxylase | 6585 | 2,4 | 14 |  | Photosynthesis | |  |
| ATP synthase | 5688 | 2,1 | 40 |  | Transport | |  |
| Cytochrome P450 | 5377 | 2,0 | 42 |  | Oxidoreductase activity | |  |
| Cytochrome b; bc1; b6f; c | 4363 | 1,6 | 41 |  | Photosynthesis | |  |
| Carbonic anhydrase | 2284 | 0,8 | 12 |  | Carbon utilization | |  |
| **Total** | **275397** | **91** | **5229** |  |  | |  |

^a^ Unitrans are blasted against UniProt (plant), having a top match with a listed gene product (BLASTx, E-value ≤1e-20).

^b^ Based on Gene Ontology (GO) database

^c^ Including “Uncharacterized protein“, „Whole genome shotgun sequence”, “Predicted protein”, “ORF”
