# Supplementary material for: An elm EST database for identifying leaf beetle egg-induced defense genes
Source: BMC Genomics. 2012 Jun 15;13:242. doi: 10.1186/1471-2164-13-242 (PMC3439254; doi:10.1186/1471-2164-13-242)
Supplement: Additional file 9 — Ipath maps. [file 1471-2164-13-242-S9.pptx]

## Slide 1
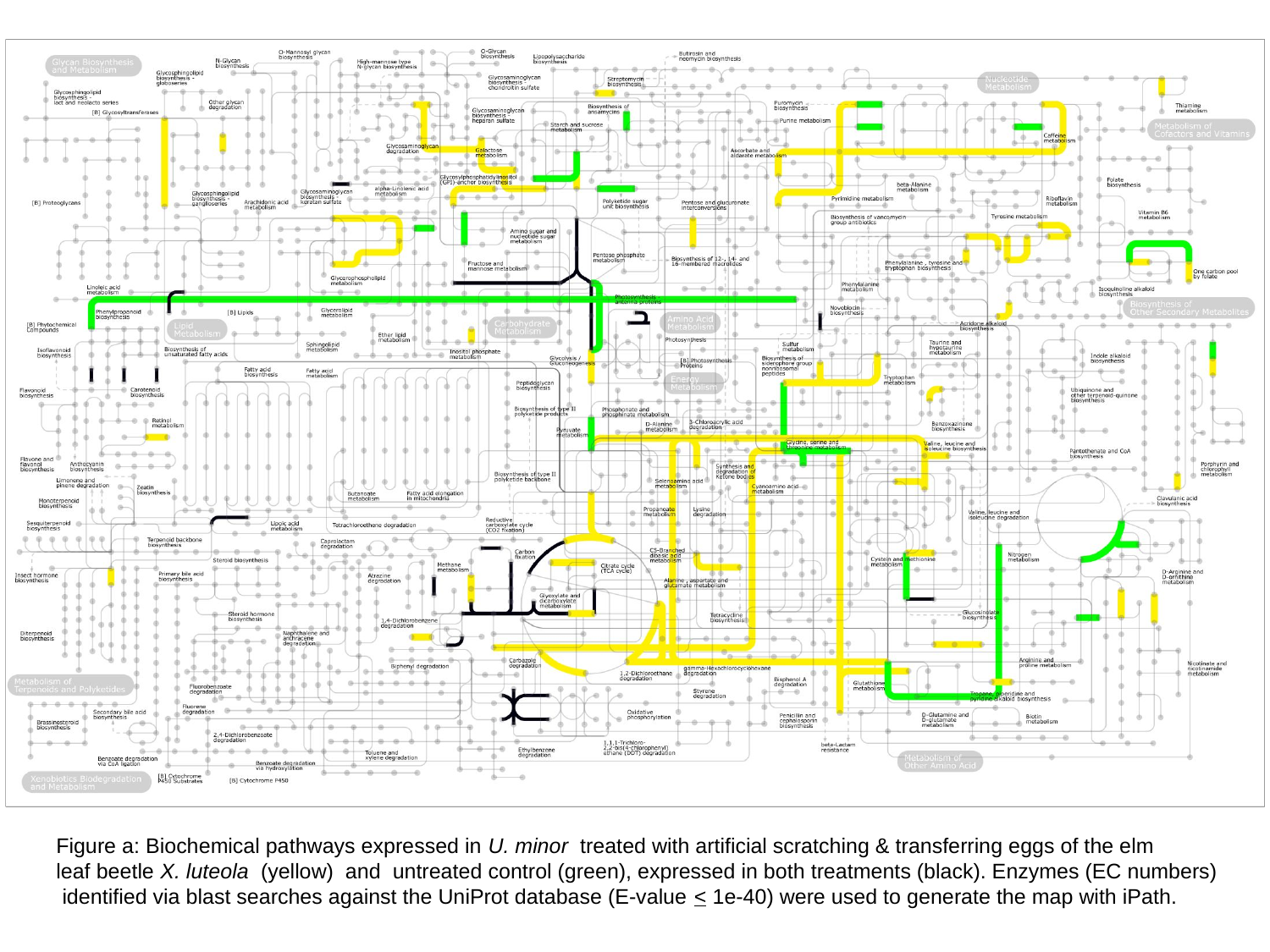

Figure a: Biochemical pathways expressed in U. minor treated with artificial scratching & transferring eggs of the elm
leaf beetle X. luteola (yellow) and untreated control (green), expressed in both treatments (black). Enzymes (EC numbers)
 identified via blast searches against the UniProt database (E-value < 1e-40) were used to generate the map with iPath.

## Slide 2
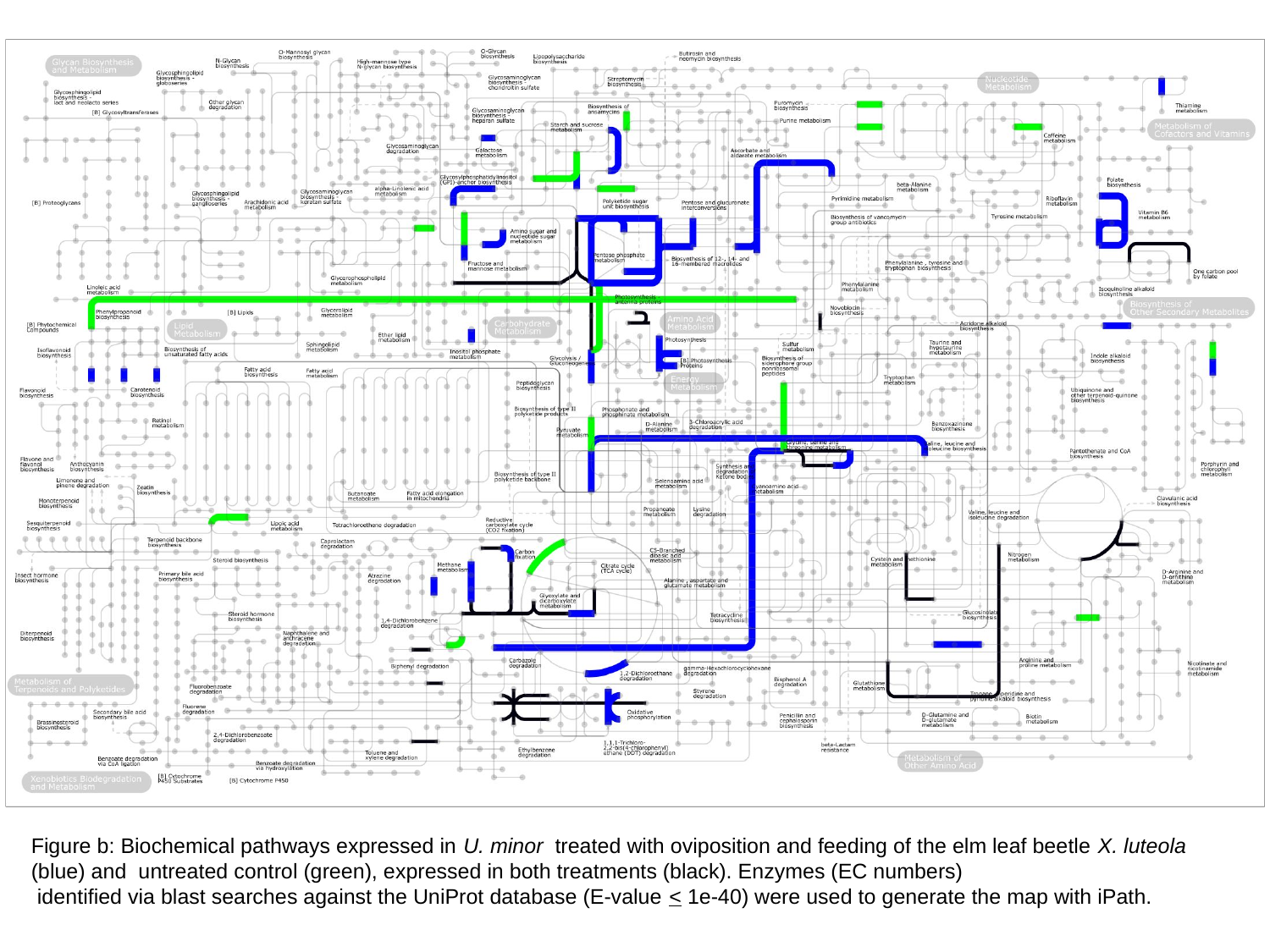

Figure b: Biochemical pathways expressed in U. minor treated with oviposition and feeding of the elm leaf beetle X. luteola
(blue) and untreated control (green), expressed in both treatments (black). Enzymes (EC numbers)
 identified via blast searches against the UniProt database (E-value < 1e-40) were used to generate the map with iPath.

## Slide 3
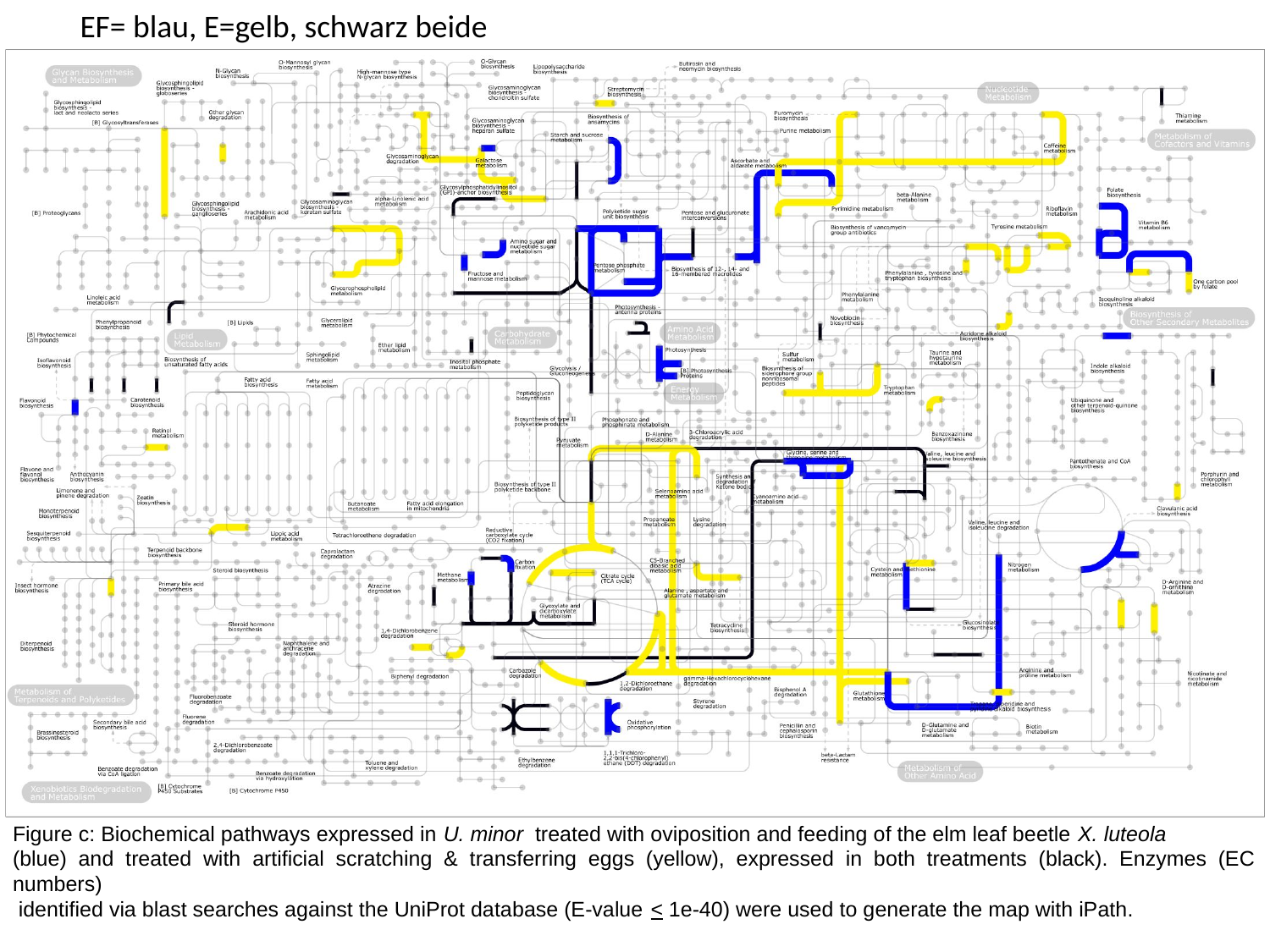

EF= blau, E=gelb, schwarz beide
Figure c: Biochemical pathways expressed in U. minor treated with oviposition and feeding of the elm leaf beetle X. luteola
(blue) and treated with artificial scratching & transferring eggs (yellow), expressed in both treatments (black). Enzymes (EC numbers)
 identified via blast searches against the UniProt database (E-value < 1e-40) were used to generate the map with iPath.

## Slide 4
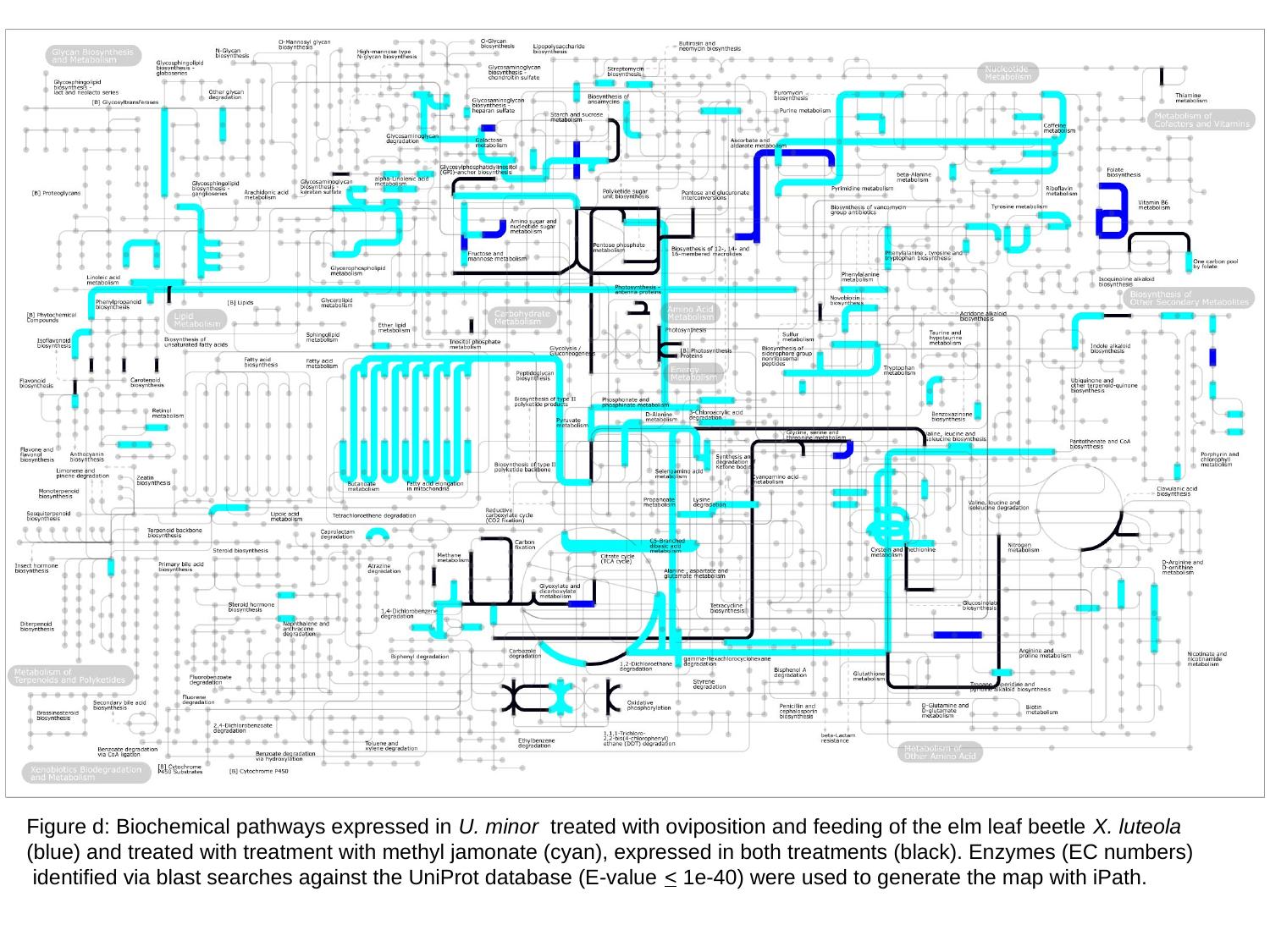

#
Figure d: Biochemical pathways expressed in U. minor treated with oviposition and feeding of the elm leaf beetle X. luteola
(blue) and treated with treatment with methyl jamonate (cyan), expressed in both treatments (black). Enzymes (EC numbers)
 identified via blast searches against the UniProt database (E-value < 1e-40) were used to generate the map with iPath.

## Slide 5
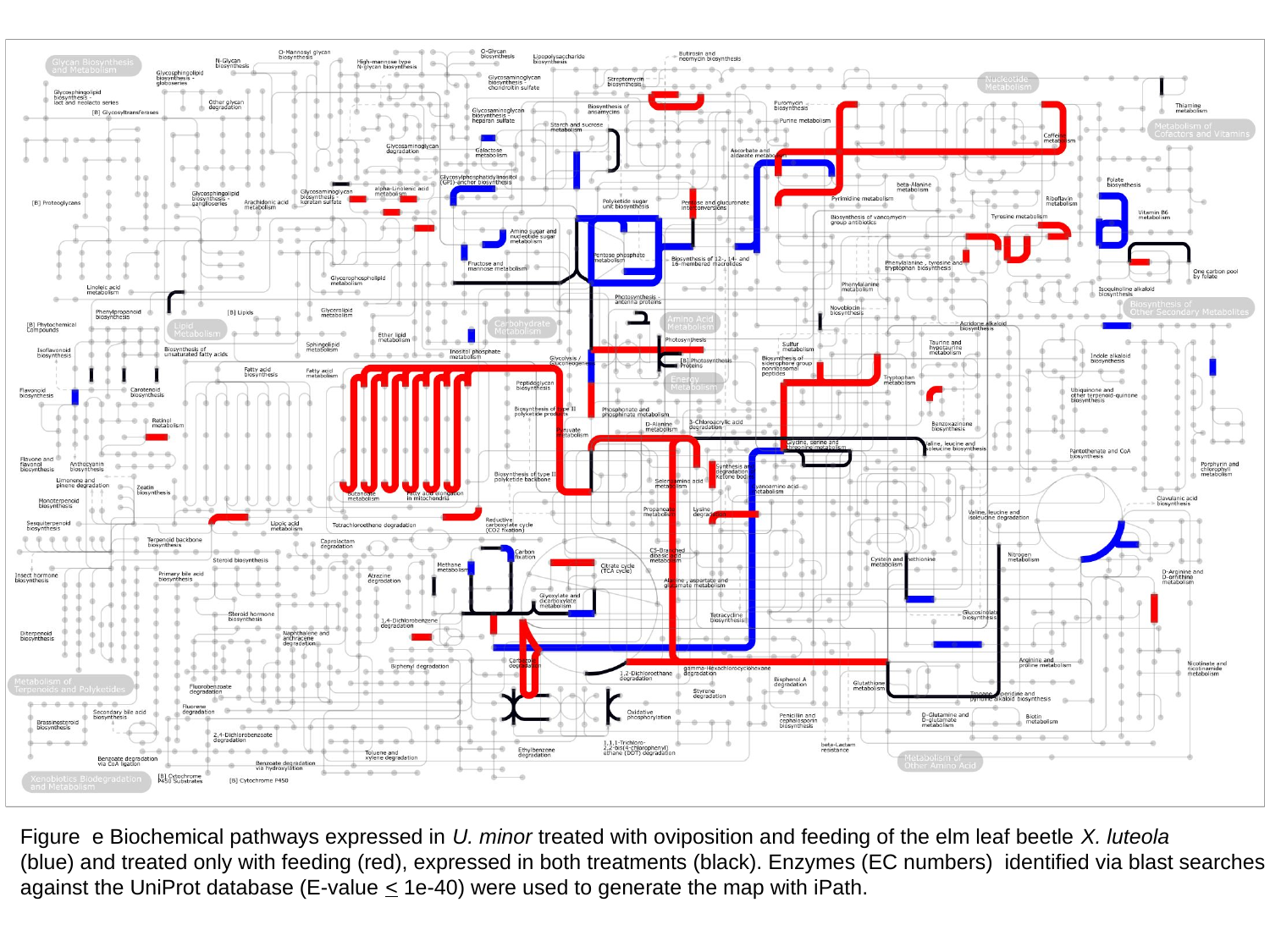

Figure e Biochemical pathways expressed in U. minor treated with oviposition and feeding of the elm leaf beetle X. luteola
(blue) and treated only with feeding (red), expressed in both treatments (black). Enzymes (EC numbers) identified via blast searches
against the UniProt database (E-value < 1e-40) were used to generate the map with iPath.

## Slide 6
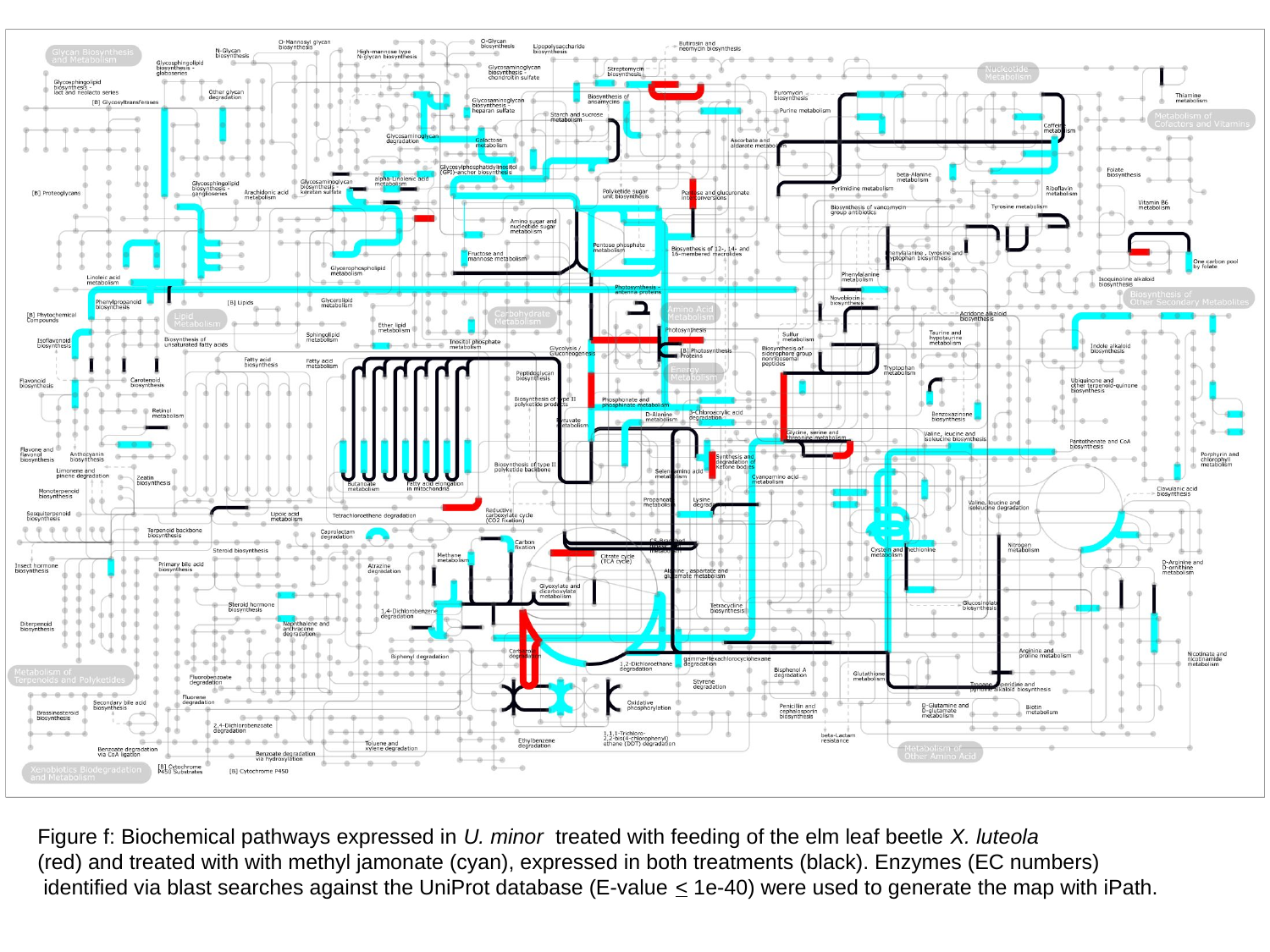

Figure f: Biochemical pathways expressed in U. minor treated with feeding of the elm leaf beetle X. luteola
(red) and treated with with methyl jamonate (cyan), expressed in both treatments (black). Enzymes (EC numbers)
 identified via blast searches against the UniProt database (E-value < 1e-40) were used to generate the map with iPath.

## Slide 7
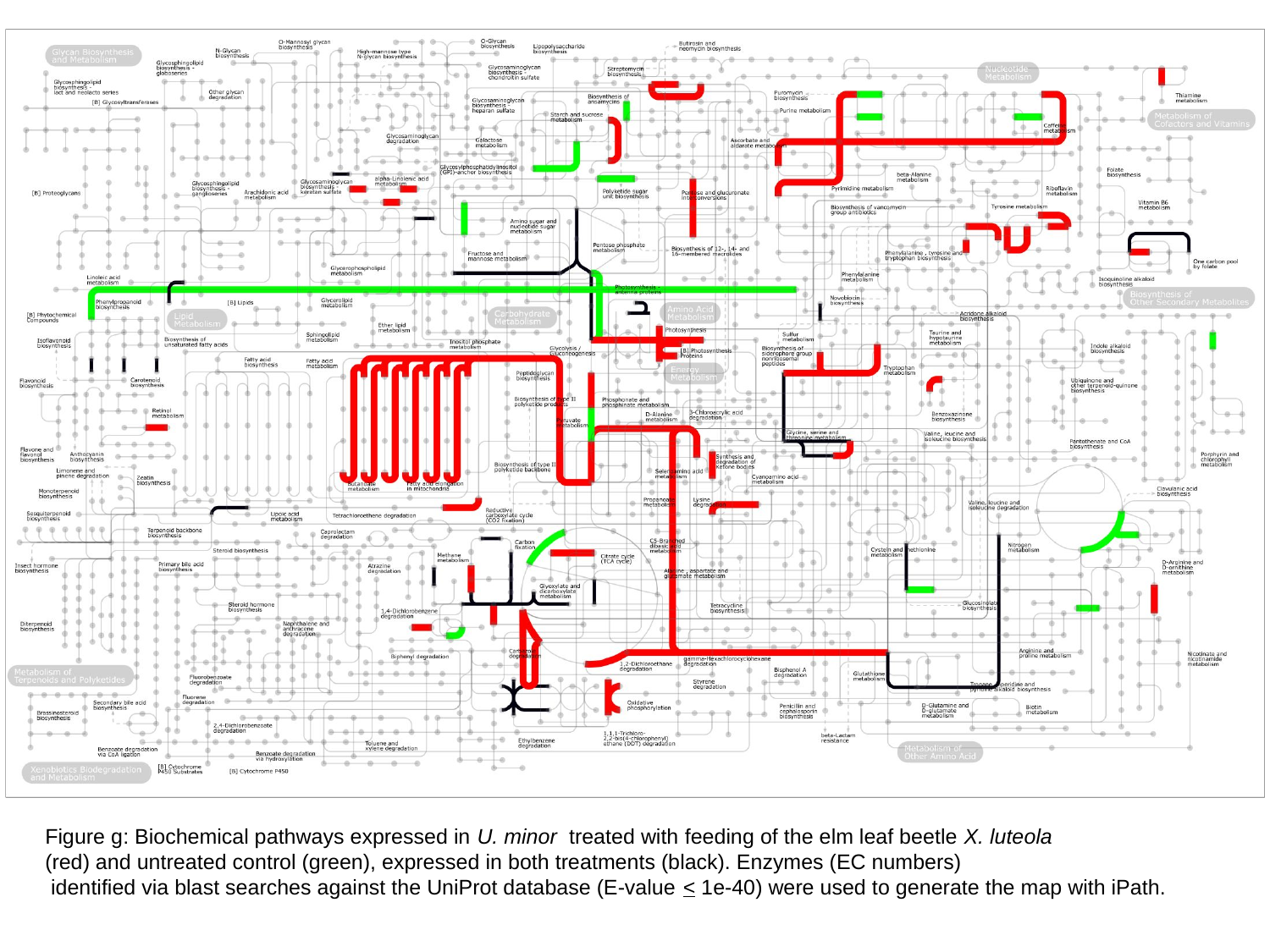

Figure g: Biochemical pathways expressed in U. minor treated with feeding of the elm leaf beetle X. luteola
(red) and untreated control (green), expressed in both treatments (black). Enzymes (EC numbers)
 identified via blast searches against the UniProt database (E-value < 1e-40) were used to generate the map with iPath.

## Slide 8
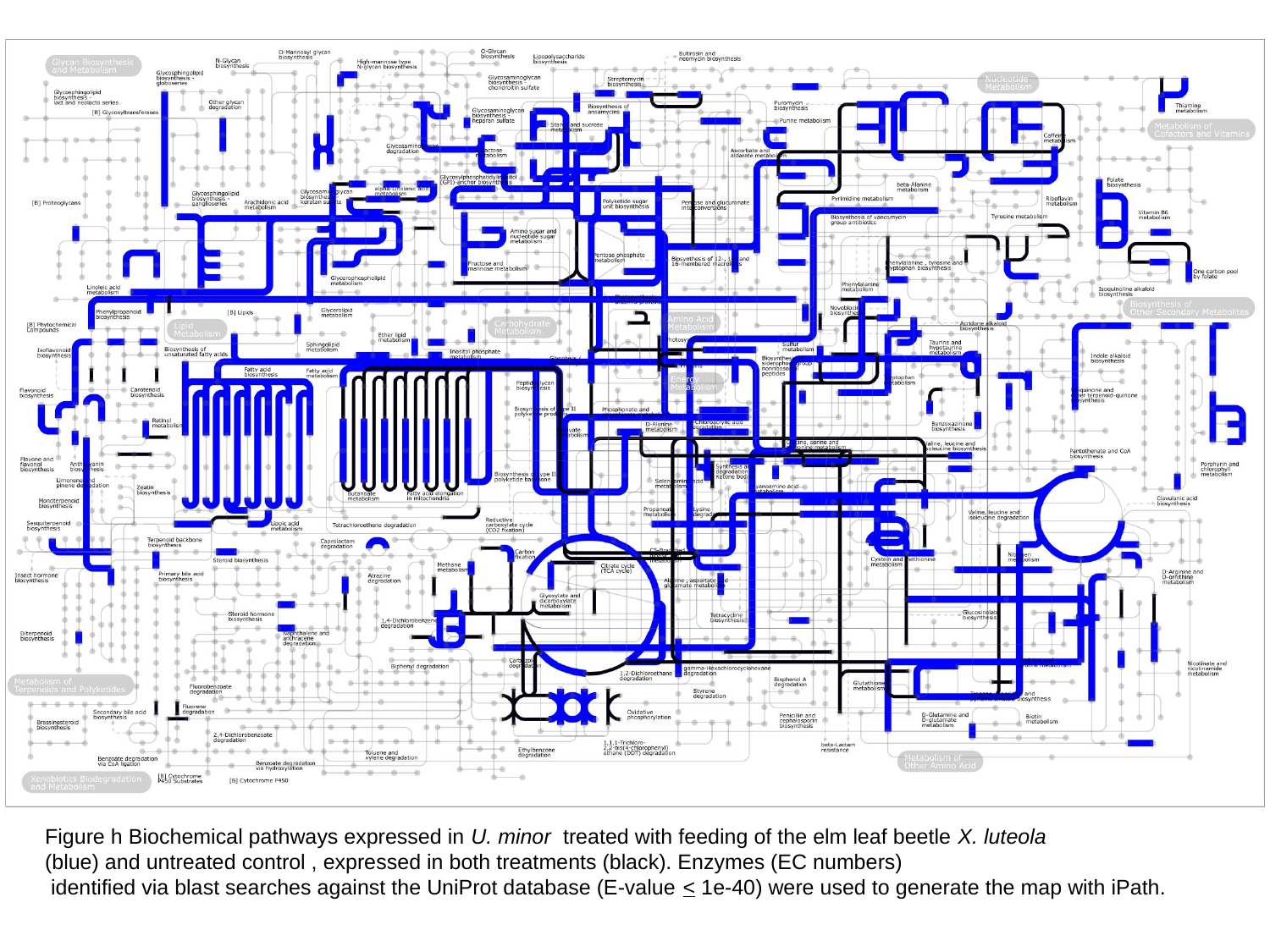

Figure h Biochemical pathways expressed in U. minor treated with feeding of the elm leaf beetle X. luteola
(blue) and untreated control , expressed in both treatments (black). Enzymes (EC numbers)
 identified via blast searches against the UniProt database (E-value < 1e-40) were used to generate the map with iPath.

## Slide 9
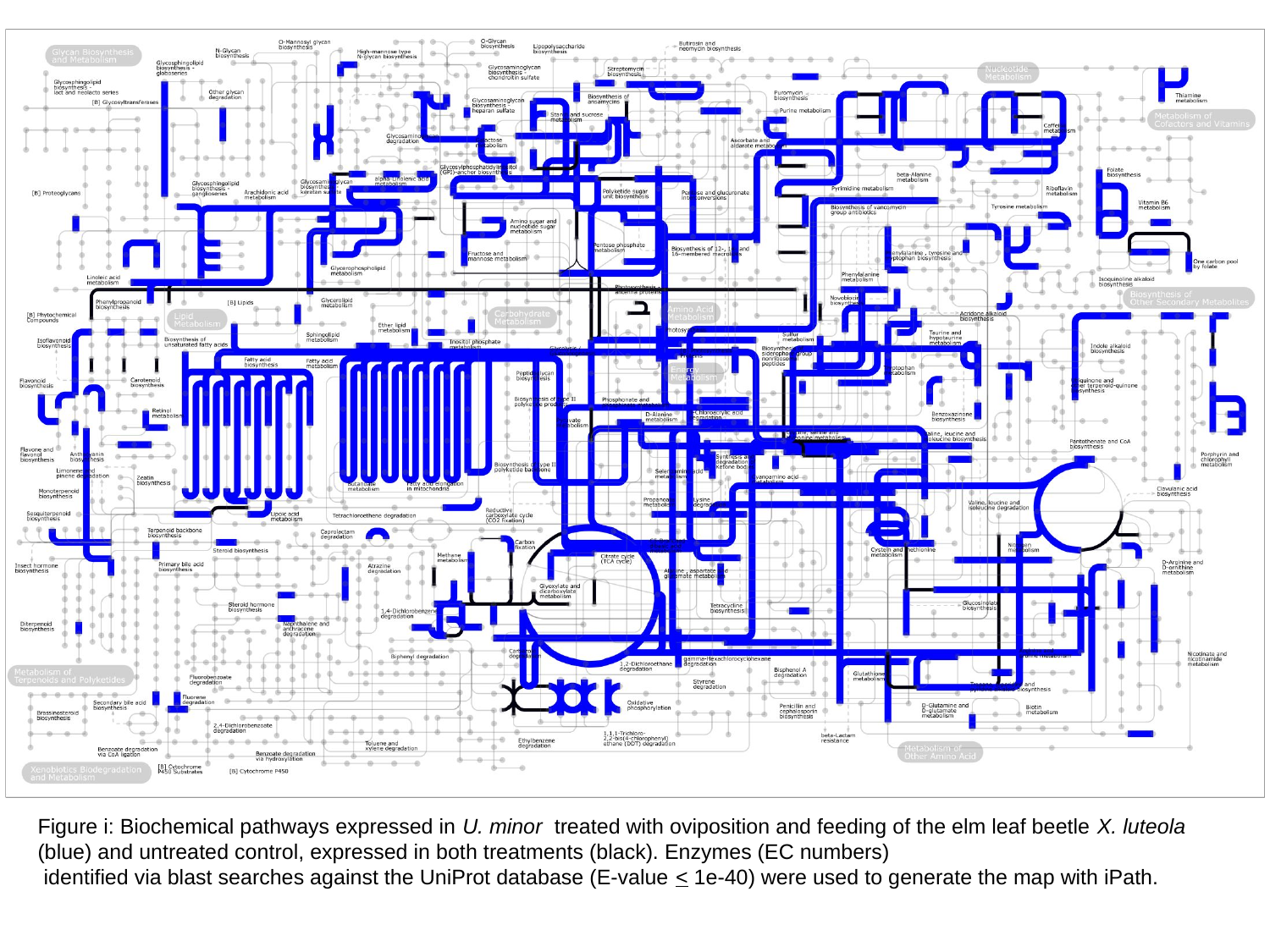

Figure i: Biochemical pathways expressed in U. minor treated with oviposition and feeding of the elm leaf beetle X. luteola
(blue) and untreated control, expressed in both treatments (black). Enzymes (EC numbers)
 identified via blast searches against the UniProt database (E-value < 1e-40) were used to generate the map with iPath.
